# Transcranial alternating current stimulation (tACS) at gamma frequency: an up-and-coming tool to modify the progression of Alzheimer’s Disease

**DOI:** 10.1186/s40035-024-00423-y

**Published:** 2024-06-27

**Authors:** Maria Luisa De Paolis, Ilaria Paoletti, Claudio Zaccone, Fioravante Capone, Marcello D’Amelio, Paraskevi Krashia

**Affiliations:** 1 Department of Medicine and Surgery, Università Campus Bio-Medico Di Roma, Via Alvaro del Portillo, 21 – 00128 Rome, Italy; 2 Fondazione Policlinico Universitario Campus Bio-Medico, Via Alvaro del Portillo, 200 – 00128 Rome, Italy; 3 Department of Experimental Neurosciences, IRCCS Santa Lucia Foundation, Via del Fosso Di Fiorano, 64 – 00143 Rome, Italy; 4 Department of Sciences and Technologies for Sustainable Development and One Health, Università Campus Bio-Medico Di Roma, Via Alvaro del Portillo, 21 – 00128 Rome, Italy

**Keywords:** Alzheimer’s disease, Mild cognitive impairment, Non-invasive brain stimulation, Neuromodulation, Brain waves, Gamma oscillations, Parvalbumin interneurons, Network dysfunction, Early intervention, Theranostic approach

## Abstract

The last decades have witnessed huge efforts devoted to deciphering the pathological mechanisms underlying Alzheimer’s Disease (AD) and to testing new drugs, with the recent FDA approval of two anti-amyloid monoclonal antibodies for AD treatment. Beyond these drug-based experimentations, a number of pre-clinical and clinical trials are exploring the benefits of alternative treatments, such as non-invasive stimulation techniques on AD neuropathology and symptoms. Among the different non-invasive brain stimulation approaches, transcranial alternating current stimulation (tACS) is gaining particular attention due to its ability to externally control gamma oscillations. Here, we outline the current knowledge concerning the clinical efficacy, safety, ease-of-use and cost-effectiveness of tACS on early and advanced AD, applied specifically at 40 Hz frequency, and also summarise pre-clinical results on validated models of AD and ongoing patient-centred trials.

## Background

Alzheimer’s Disease (AD) is an irreversible and progressive neurodegenerative disorder, with a devastating impact on the quality of life of patients and their caregivers. The staggering number of people affected with AD is about 50 million, which is expected to triple by 2050, causing a huge burden on healthcare systems [1, 2]. AD is the most common form of dementia and is characterized by a progressive loss of memory, deficits in cognitive functions (e.g., word retrieval, language comprehension, visuospatial orientation, abstract thinking and judgment) and neuropsychiatric symptoms (e.g., mood disorders, apathy, anxiety, irritability, aggression and sleep disturbances), with the latter often appearing years before the cognitive decline [3–6].

The amyloid cascade has been considered the main explanation for AD pathogenesis for the last 40 years [7–10], speculating that neuronal loss depends on the toxic nature of amyloid β (Aβ) soluble oligomers, fibrils or plaques. According to this hypothesis, an increased production of the toxic Aβ_42_ fragment, following the cleavage of amyloid precursor protein (APP) by β- and γ-secretases, leads to enhanced oligomerisation and fibril formation compared to the Aβ_40_ fragment, and to fast accumulation of these fibrils into plaques, exacerbating the neurotoxicity. Indeed, Aβ oligomerisation, fibrillogenesis, aggregation and deposition into plaques spark a deleterious process that leads to synaptic degradation, neuronal loss, neurodegeneration and neuroinflammation. The discovery of genetic mutations (*APP*, *PSEN1*, and *PSEN2*) and the genetic risk factor (*APOE* ε4 allele) has corroborated the amyloid cascade hypothesis. Indeed, Aβ pathology still remains the most common target in clinical trials for AD treatment [11] and, throughout the years, many anti-Aβ monoclonal antibodies with specific biochemical targets have been developed to block its cascade at different stages. Despite the failure of many potential candidates [12, 13], this effort resulted in the recent FDA approval of two anti-Aβ antibodies, aducanumab and lecanemab, based on imaging data showing reduction in amyloid burden and clinical results indicating a slowing-down of cognitive decline and improvement in daily living [14–16]. Yet, these drugs are also associated with adverse events such as amyloid-related imaging abnormalities (ARIA) and variable changes in brain volume, that, in the case of aducanumab, have raised important issues regarding the risk–benefit profile and have led to the refusal by the European Medicines Agency to authorise the drug [17, 18]. On the other hand, interesting results on safety are to be expected from the ongoing trial on lecanemab in healthy young individuals with intermediate or elevated amyloid levels at risk of developing AD ([19] – AHEAD trial NCT04468659).

A key factor contributing to the failure of many clinical trials can be attributed, in part, to their late administration in the disease course. Indeed, the timing of the therapeutic intervention plays a critical role. As a matter of fact, many years before clinical diagnosis, most disease-related processes are already active in destroying synapses and neuronal connections [20]. These alterations occur even before the mild cognitive impairment (MCI)-due-to-AD phase, the transitional period between normal ageing and the diagnosis of clinically-probable very-early AD [21]. The annual conversion rate from MCI to dementia ranges from approximately 22% (community-based studies) to 39% (memory clinics), with most individuals developing AD [22]. Thus, in the context of AD, the MCI phase represents a unique and crucial time-window for intervention to allow the slowing-down of the disease progression. For this reason, specific and accurate diagnosis of MCI-due-to-AD is essential in order to apply tailored treatments, since the “one-size-fits-all” approach may not be suitable because of the various disease trajectories observed in MCI patients.

Indeed, in the MCI and early-AD stages, patients and AD animal models show neuronal hyperexcitability, spontaneous epileptiform activity and changes in cerebral network oscillations [23–25]. In particular, reductions in alpha (7–13 Hz)-, beta (14–30 Hz)-, and/or gamma (25–140 Hz)-frequency bands, and increases in the theta (4–7 Hz) band have been described [26, 27]. A particular interest currently is on gamma oscillations, which act physiologically as a “binding factor” that coordinates the activities of various neurons in brain areas involved in complex cognitive functions. When gamma wave dysregulation occurs, network coordination fails, contributing to the cognitive deficits seen in MCI ([26, 28, 29]; BOX 1).

In this conceptual framework of conceiving AD as a circuit-based disorder, a new therapeutic paradigm is hypothesized: is it possible to slow down or block disease progression by tuning up these precocious neural circuit alterations? Non-invasive brain stimulation (NIBS) techniques represent a promising intervention tool, not only for their tolerability and safety, but also because they can be used to directly target specific neural circuit disturbances (see [30–32] for thorough reviews). In fact, the challenges related to current drug treatments (such as the potential ineligibility of patients, the absence of clinically-relevant cognitive improvements after therapy, the high costs or potential side effects) are pushing the scientific community to explore new and alternative non-pharmacological approaches for AD treatment. Among the NIBS techniques, transcranial alternating current stimulation (tACS) is receiving particular interest in the AD field due to its relationship with brain oscillations. In this review, we aim to discuss the current understanding of this promising therapeutic strategy, with a major focus on tACS at 40 Hz gamma frequency, as a potential way to restore the disrupted brain connectivity and potentially mitigate AD symptomatology.

## Unravelling the potential of tACS in neurostimulation: mechanisms and insights

Among the different NIBS methods, transcranial electrical stimulation represents a group of techniques based on the delivery of weak electric currents to modulate neural activity in the brain. This versatile method includes several procedures that, depending on the way electric current is delivered, can be classified as continuous (transcranial direct current stimulation, tDCS), random (transcranial random noise stimulation, tRNS) or alternating (tACS), each resulting in distinct neurostimulation effects. Among these methods, tACS stands out as a cutting-edge approach, representing a significant advance in the field of NIBS because it offers a unique and precise protocol to tune up the brain’s intrinsic oscillations, opening up new possibilities for personalized brain stimulation and therapeutic interventions in various neurological and psychiatric conditions, including AD.

### Mechanism of action—how tACS works

tACS represents a simple procedure based on the delivery of low-intensity electric current (in humans it typically ranges from 1 to 4 mA) by the application of two or more electrodes connected to a battery-powered current stimulator [32]. The electrodes, strategically placed on the scalp over the targeted brain region, deliver alternating currents which penetrate the skull and, at the selected frequencies and intensities, can modulate the electrical activity of neurons in the underlying brain areas, for a selected treatment duration. More specifically, the current delivered moves from the positive maximum pole to the negative maximum one, providing a single repeating cycle in sinusoidal motion with a specific frequency.

As the current penetrates the skull and reaches the targeted brain region, it induces alterations of the membrane potential towards depolarization or hyperpolarization in an oscillatory fashion both in cell bodies and in dendrites of neurons [33]. This alternating change in the membrane potential is considered sufficient to change the neuronal probability of generating action potentials [34]. However, it does not directly affect the firing rate of action potentials; instead, it governs their timing in a frequency- and location-specific manner [35]. The effectiveness of tACS is influenced not only by the amplitude and frequency of the stimulation but also by the three-dimensional orientation of both neurons and the penetrating current. Its effects stem from the modification of the membrane potential of neurons aligned with the induced electric field [36]. Consequently, when these cells are stimulated by tACS, they exhibit specific frequency resonance, long-lasting after-effects and long-distance oscillatory connectivity with their targets. Moreover, the orientation of the electric stimulation significantly alters the characteristics of the resulting field and, consequently, its impact on neurons [37].

While the precise mechanisms underlying the action of tACS remain not fully understood, electrophysiological approaches, primarily electroencephalography (EEG) or magnetoencephalography in humans, as well as intracranial recordings and local field potential recordings in animals have been employed to investigate tACS mechanisms [33, 38–40]. Such studies have identified two main categories of effects: online effects, which can help assess phase modulation during stimulation, as well as offline effects, which can help assess the lasting effects of stimulation, after stimulation has ended. It has been suggested that the online effects involve entrainment of brain oscillations to the stimulation frequency and coupling or decoupling of long-range oscillatory connectivity between distant brain regions. Yet, the offline effects more likely involve spike-timing dependent plasticity (STDP) rather than entrainment [40–42]. These two mechanisms [43] have been proposed as the main putative mechanisms of action of tACS. Entrainment is a phenomenon based on the synchronization of an oscillating unit to an external driving force. The interaction between the internal oscillator and the external driving force is unidirectional; thus, only the internal oscillations are influenced by the external driving force and not *vice versa*. Entrainment is thought to be most effective when the stimulating frequency is at or close to the endogenous frequency of the targeted area, and when the endogenous oscillations approximate the external driving force, both rhythms become coupled [44]. In STDP, the stimulation leads to synaptic changes based on the timing of neuronal firing. Synaptic strength increases when pre-synaptic spikes occur prior to the post-synaptic spikes (long-term potentiation, LTP). Conversely, when post-synaptic spikes occur prior to pre-synaptic spikes, synapses weaken (referred to as long-term depression, LTD; [45]). This tool produces periodic changes in the firing frequency of the targeted neuronal networks and consequentially can enhance or reduce the magnitude of continuous physiological oscillatory rhythms [46–48]. Despite this knowledge, the extent at which entrainment and plasticity events produced by tACS are interconnected, or whether their effects are independent, remains to be defined.

Another important aspect related to the efficacy of tACS is the intensity-effect relationship. When administered at a relatively low intensity, neuronal entrainment is relevant to only a few cells, but application of higher current intensities, considered safe for humans, results in an increase of the electric fields and in a consequent higher number of neurons entrained by tACS. This suggests the existence of a minimum effective intensity [35, 49, 50]. The direct relationship between entrainment and current intensity provides evidence for the dose-dependent impact of tACS on neuromodulation. Yet, this relationship is not strictly linear as it was shown that weaker stimulation intensities, below the minimum effective one, induces de-entrainment of neurons to the endogenous oscillation instead of the external one [51, 52]. These and other experiments represent an important basis for choosing the most appropriate parameters for translational applications of tACS in humans.

### Safety considerations

Working at very low intensities, tACS appears to be a non-invasive, risk-free intervention, with only modest and temporary side effects that are typically well tolerated. These include moderate headache, nausea, fatigue and skin reactions such as itching, tingling and redness under the electrodes used to administer the current [53–55]. However, although tACS is generally considered safe when applied within the recommended parameters, it is essential to consider individual differences in brain anatomy and electrical conductivity to avoid adverse effects. Careful electrode placement, choice of stimulation intensity and monitoring are necessary to minimize potential risks.

The paucity of important side effects represents a comparative advantage of tACS over anti-amyloid monoclonal antibody therapies and can be a salient point of consideration within the realm of AD therapeutics. Indeed, the new monoclonal antibody treatments, while promising in their ability to target the underlying amyloid pathology, come with strict eligibility criteria that limit their applicability to only a small percentage of patients in the early stages of AD. This is what emerged from a recent publication that shed light on how the eligibility of early-AD patients for treatment with aducanumab or lecanemab is actually limited due to exclusionary common conditions of the typical older adult population (including cognitive performance and age, body mass index, comorbidities, brain microhaemorrhage suspicious for co-morbid angiopathy [56]). Of the 237 participants with MCI or mild dementia and increased cerebral amyloid load, only 47% met the inclusion criteria for lecanemab’s trial, reducing to 8% after exclusion criteria were added. For aducanumab, only 44% of patients were eligible, falling to 5% after exclusion criteria were applied. Conversely, the non-invasive nature of tACS alleviates concerns pertaining to systemic adverse effects and procedural complications, rendering it potentially suitable across a broader demographic spectrum, which is particularly relevant in the context of AD patients, typically characterised by variable clinical profiles and medical histories. It is also important to note that the individualised dosimetry and customisable nature of tACS parameters allow for tailored interventions, taking into account inter-individual variability in neurophysiological responses and treatment requirements. This approach ensures optimal efficacy while minimising the risk of adverse events, thus enhancing the overall safety profile of tACS and circumventing the risk–benefit dilemmas associated with pharmacological agents in the context of AD treatment.

Additionally, the integrative potential of tACS within the existing treatments for AD also holds significant promise. By virtue of its mechanism of action, tACS may augment the efficacy of the current anti-amyloid therapies, thereby offering a multifaceted approach to disease management. Continued advancements in neuroimaging techniques, coupled with a deeper understanding of the precise neurobiological mechanisms underlying tACS-mediated effects, are poised to further refine treatment strategies and optimize clinical outcomes in AD patients. Thus, the relative absence of side effects for tACS administration in AD patients engenders optimism for its adjunctive or standalone use alongside the approved anti-amyloid therapies. At the same time, as often occurs with other multi-therapeutic approaches, the side effects of the single treatments can compound each other, leading to an increased risk of harm. For example, nausea and headache are two of the common side effects of both lecanemab and aducanumab [14, 16], and they may also be experienced by patients during tACS sessions. Therefore, it should be noted that there is a likelihood of an increase in the risk of both nausea and headache when tACS is administered to individuals undergoing concurrent monoclonal treatment, despite these side effects being considered minor.

In comparison, a more infrequent but more dangerous side effect of monoclonal antibodies is ARIA, that, even if present more often asymptomatically, can lead to permanent invalidity and death [57]. In particular, two major subtypes of this condition have been described: ARIA-oedema/effusion (ARIA-E) and ARIA-haemosiderosis/microhaemorrhages (ARIA-H), both of which appear to depend on a disruption in vascular permeability of the blood–brain barrier, leading to an overflow of fluid in the subpial space (ARIA-E) and in worst cases of erythrocytes (ARIA-H), resulting in an inflammatory reaction and haemoglobin-dependent damage. In this scenario, the simultaneous administration of a monoclonal antibody and of tACS, among whose effects is an increase in vascular permeability [58], might result in a higher risk of developing ARIA, raising the risk of morbidity and mortality. For this reason, it is necessary to further evaluate the potential synergistic effect of both monoclonal therapy and tACS in increasing the risk of side effects. Precise selection of patients who can receive greater benefit from the combination of these two lines of therapy with less risk is also important.

### Frequency-specific effects

Recent research has highlighted the importance of frequency-specific effects of tACS. Different brain regions and cognitive processes are associated with distinct frequency bands. These frequencies dominate the EEG recordings when the brain is performing specific functions in the awake or different sleep states. For example, slow-wave sleep or drowsiness generates delta (1–4 Hz) and theta (4–7 Hz) band oscillations, while awake states bring instead alpha (7–13 Hz) and beta waves (14–30 Hz) that support wide-ranging neural processes including voluntary movement [59–61].

There are also brain oscillations in the gamma range (∼25–140 Hz) which are related to several cognitive functions, including working memory, learning and attention [62, 63]. It is common to observe an alteration of this oscillatory pattern in disorders affecting memory and cognition, such as AD (BOX 1), and for this reason gamma-frequency tACS caught particular attention as it can influence perceptual and memory processes [61, 62]. tACS can effectively “tune up” the brain’s endogenous neural oscillations and modulate their activity in a targeted manner. Indeed, when the external current frequency matches the endogenous brain oscillatory rhythm, neuronal spiking can be amplified or re-established. Specifically, as mentioned above, the oscillating currents can influence the membrane potential of neurons, increasing the probability that neurons generate synchronized action potentials, crucial for proper information processing [32]. Understanding how the timing and phase of tACS interact with the brain’s intrinsic rhythms is therefore crucial for optimizing stimulation protocols and improving their efficacy.

### 40 Hz – The neuroenhancement frequency

Gamma waves are reduced in both MCI and AD patients [26, 64–66], as well as in various AD mouse models [67–70]. The etiopathogenesis of gamma wave alterations has not yet been clarified, although the involvement of deficits in parvalbumin-expressing GABAergic interneurons (PV-INs) is certain (BOX 2).

**Fig. 1:**
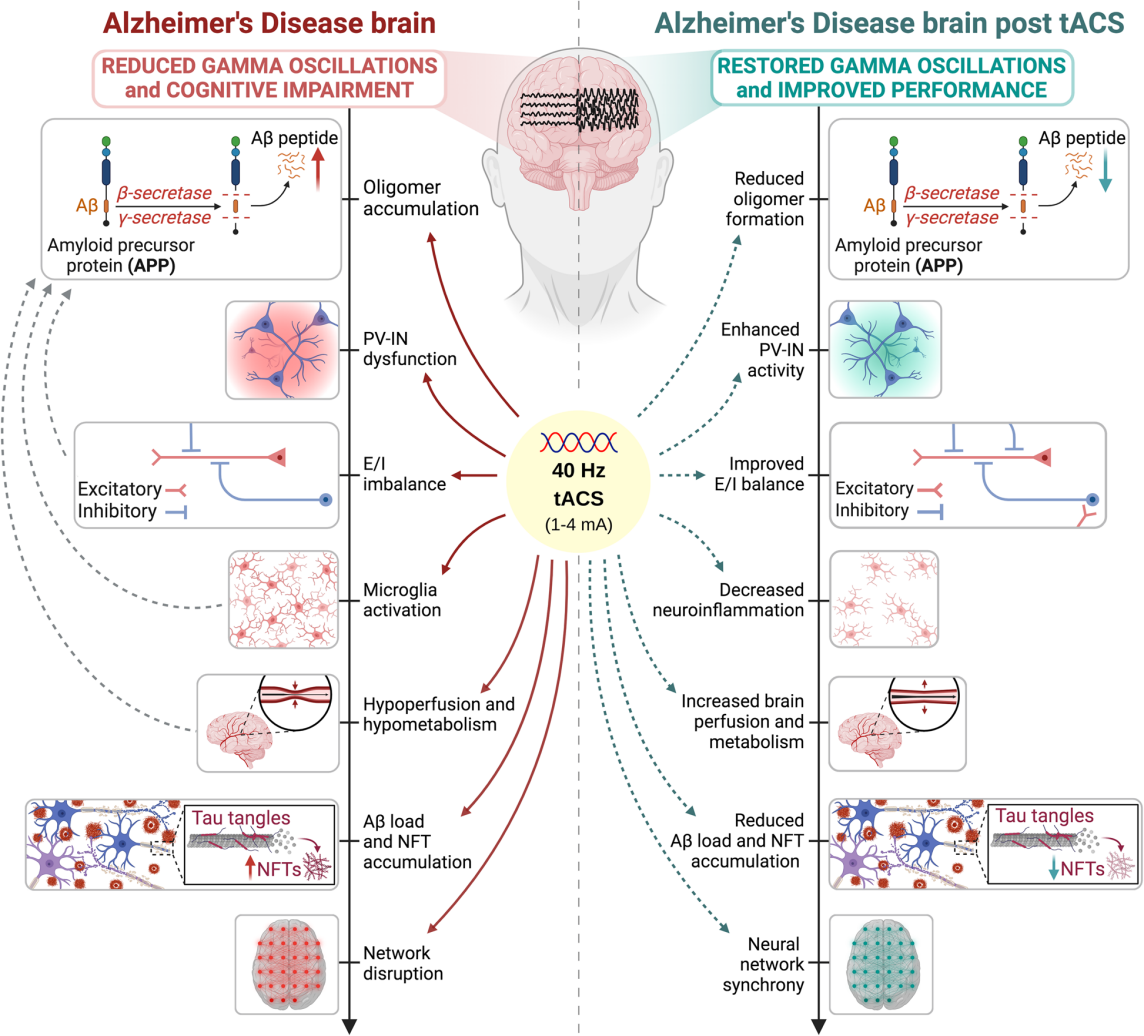
40 Hz tACS as a potential therapy for AD. The left side of the figure illustrates the untreated, diseased state. The AD brain is marked by compromised gamma frequency oscillations and cognitive deficits. The pathological sequence commences with the excessive production of Aβ soluble oligomers, resulting in PV-IN dysfunction and subsequent dysregulation of the excitation (E) / inhibition (I) balance. The presence of excessive Aβ and the neuronal dysregulation instigate neuroinflammatory responses, leading to cerebral hypoperfusion and consequent brain hypometabolism. The interplay among E/I imbalance, neuroinflammation and hypoperfusion exacerbates Aβ oligomerization (indicated by gray dashed arrows), promoting a self-perpetuating cycle culminating in plaque and neurofibrillary tangle (NFT) formation. The pathological cascade reaches its peak with network disruption, characterized by the broad term “oscillopathy”. Conversely, the right side depicts the therapeutic potential of 40 Hz tACS. Diverse tACS protocols can be tailored to specific frequencies and electrode placements, contingent upon the targeted neural wave synchronization. Preliminary pre-clinical and clinical trials suggest that 40 Hz tACS restores the amplitude of gamma oscillations, synchronizing PV-IN activity and restoring E/I balance. Consequently, this can mitigate neuroinflammation, enhance cerebral perfusion and metabolism and facilitate a decrease in oligomer formation, overall reducing Aβ burden and tangle accumulation. Ultimately, interrupting the pathological cycle would lead to the reinstatement of neural network synchrony and cognitive enhancement. Red solid arrows denote the levels at which 40 Hz tACS is presumed to intervene to address the aforementioned pathological mechanisms, while green dashed arrows signify the improvements that can be elicited by tACS across the same pathological processes

Among the different frequencies of the gamma band (ranging from 25 to 140 Hz), the most used in AD is the gamma stimulation at 40 Hz. The reason of choosing such frequency in AD treatment is based on a pioneer study observing that modulation of gamma activity with optogenetic stimulation at 40 Hz frequency of PV-INs in 5 × FAD mice increases neuroprotective factors, preserves cortical thickness and spine density, slows down neuronal death, decreases amyloid levels, microgliosis and inflammatory gene expression, and improves performance in memory and cognition tests [69]. Subsequent pre-clinical studies showed that non-invasive 40 Hz stimulation with sensory stimuli (named "gamma entrainment using sensory stimuli", or GENUS) led to comparable outcomes by the same and by other research groups, also in other mouse models [71–79]. These promising molecular and behavioural improvements in AD mice inspired researchers worldwide to use 40 Hz as a key frequency to restore the gamma oscillopathy typical of AD. In fact, in the last years, several important clinical trials have used 40 Hz gamma modulation to investigate its therapeutic potentialities (for reviews see [80–83]), with most works focusing on sensory stimulation. Yet, the interest is also increasing for the usage of 40 Hz tACS (Fig. 1).

Of note, although the gamma frequency appears to represent a key frequency range to stimulate memory function, especially for AD patients, gamma tACS can also target neural oscillations occurring in a wider scale of dysfunctional networks, such as the hippocampal-prefrontal circuit. Considering the hippocampus and its role in short- and long-term memory processing, close attention should be paid also to theta waves (4–8 Hz), in parallel to gamma waves, as both are required to encode memory and perform cognitive abilities [84]. Oscillations in gamma and theta bands occur simultaneously, and high-frequency gamma oscillations have been shown to be modulated by the low-frequency theta oscillations. This type of interaction between waves and cross-frequency coupling (also known as phase-amplitude modulation; [61, 85]) reflects integration of neural code across different brain areas or gating information between populations of neurons [86]. Interestingly, it has been highlighted that the study of goal-oriented behavior and cognitive function should not rely on single brain regions but on the analysis of neuronal oscillations in different distributed neural circuits. This serves to extrapolate “fingerprints” of oscillating correlations occurring in cortical networks involved in cognitive processing [87]. The value of this approach is further evidenced by the idea that segmentation of different types of oscillations and rhythmic activity may serve to separate temporal aspects of memory (i.e., ordering of events as they appear in action) and in particular of working memory such as for the phases of encoding, maintenance and retrieval [88].

Here, despite the critical relevance of theta-gamma cross-frequency coupling for short- and long-term memory, we will take into consideration only gamma band stimulation, by particularly highlighting pre-clinical, clinical research and ongoing clinical trials that employed 40 Hz tACS as a potential therapy method for AD (for a summary see also Table 1).

**Table 1:** Summary of clinical studies investigating the effects of 40 Hz tACS in MCI and AD patients

Authors, journal publication year (*clinical trial*)	Experimental group	Type of stimulation	Treatment duration	Electrode Placement	Assessments	Outcomes	Side Effects
Altomare et al., 2023 *(PNRR-POC-2022–12376021)* [127]	**Phase 1:** 60 AD (divided into *2 groups*: ARM1, i.e. tACS, and ARM2, i.e. sham) **Phase 2:** both groups receiving tACS	2 mA 40 Hz HB-tACS	**Phase 1:** 8 weeks, 5 days/week, single 60-min session/day **Phase 2:** 8 weeks, 5 days/week, single 60-min session/day	Pads placed on the scalp over Pz and over the right deltoid muscle	- Neuropsychological assessments: CDR-SB, ADAS-Cog, face-name association test, RAVL (both immediate and delayed recall), verbal fluency, TMT, ADCS-ADL, NPI, CBI - Power spectral analysis through EEG - Blood-based biomarker detection (i.e., Aβ_42_ and Aβ_42_/Aβ_40_, p-Tau, NfL, GFAP, neurogranin) through Simoa - SAI through TMS - Structural and functional brain network connectivity through sMRI and resting-state fMRI; cerebral perfusion through perfusion-MRI (ASL) (only at T00 and T08) - Amyloid-PET imaging (only at T00 and T08) [**Testing checkpoints:** at baseline (T00), after 8 weeks (T08, end of phase 1 and beginning of phase 2), 16 weeks (T16, end of phase 2) and 24 weeks (T24, follow-up)]	N/D (Ongoing study)	N/D (Ongoing study)
Benussi et al., 2021 *(NCT04515433)* [131]	20 MCI-to-AD (divided into *2 groups*: tACS and sham)	3 mA peak-to-peak 40 Hz tACS	Two single 1-h sessions (separated by a 1-week crossover phase within subjects)	One pad placed above the medial parietal cortex and Pz and the other one over the right deltoid muscle	- SAI through TMS - Episodic-associative assessments: RAVL and FNAT (the latter evaluated during the last 20-min of tACS) [**Testing checkpoints:** In each of two sessions, at baseline (pre-stimulation) and post-intervention]	- tACS group showed better performance in RAVL (both immediate and delayed recall) and FNAT tests compared to sham group. RAVL test (delayed recall) improvement positively correlated with FNAT performance - tACS group showed restoration of SAI. Changes in SAI positively correlated with FNAT performance	No significant SE, including visual disturbances
Benussi et al., 2022 *(NCT04842955)* [136]	**Main study:** 60 early-AD (divided into *2 groups*: tACS and sham) **Supporting information studies: 1**) 12 early-AD (divided into *2 groups*: tACS and sham) **2**) 12 early-AD (divided into *2 groups*: tACS and sham)	3 mA peak-to-peak 40 Hz tACS	Two single 1-h sessions (separated by a 1-week crossover phase within subjects)	**Main study and supporting information study 1:** One pad placed above the medial parietal cortex and Pz and the other one over the right deltoid muscle **Supporting information study 2:** One pad placed on the scalp over the right DLPFC and the other one over the right deltoid muscle	**For main study:** - SAI through TMS - Episodic-associative assessments: RAVL and FNAT (the latter evaluated during the last 20-min of tACS) - Predictors of γ-tACS Efficacy (such as *ApoE* and *BDNF* genotypes) - Power spectral analysis through resting-state EEG (in a subset of 10 randomly selected participants) **For supporting information study 1:** - Executive functions, verbal fluency, and visuospatial abilities (DS backward, phonemic, and semantic fluencies, TMT (A and B scores), CDT) **For supporting information study 2:** - Effects of tACS on stimulation site [**Testing checkpoints:** In each of two sessions, at baseline (pre-stimulation) and post-intervention]	**Main study:** - Restoration of SAI in tACS group. Changes in SAI positively correlated with RAVL (delayed recall) and FNAT performance - tACS group showed better performance in RAVL (both immediate and delayed recall) and FNAT tests compared to sham group - *ApoE* genotype was a good predictor of tACS efficacy for RAVL (both immediate and delayed recall) and FNAT; MMSE for RAVL (both immediate and delayed recall); education for FNAT - Increase in gamma and beta spectral power; decrease in theta spectral power; no differences in alpha spectral power - Increased RAVL and FNAT positively correlated with increased gamma spectral power in the parietal lobe **Supporting information study 1:** - No tACS after-effects on executive functions, verbal fluency and visuospatial abilities **Supporting information study 2:** - No tACS after-effects on the RAVLT (both immediate and delayed recall) and on FNAT scores	No significant SE, including visual disturbances (only tingling sensations)
Borroni et al. *(NCT05643326)* [128]	30 mild-AD (divided into *2 groups*: tACS and sham)	40 Hz HB-tACS	**Phase 1:** 9 weeks, 5 days/week (42 sessions) of tACS or sham stimulation (depending on the group assignment) **Phase 2 (open label):** 9 weeks, 5 days/week (42 sessions) of tACS for both groups	Pz	- Blood-based biomarker detection (i.e., Aβ_1-40_ and Aβ_1-42_, NfL, GFAP) - Amyloid-PET imaging - Functional brain network connectivity through resting-state fMRI - Assessments: CDR, ADAS-Cog, RAVLT, FNAT, NPI, ZBI, CBI - SAI through TMS - Power spectral analysis through EEG [**Testing checkpoints:** at baseline, post-intervention (9-week) and at follow-up (18- and 26-week)]	N/D (Ongoing study)	N/D (Ongoing study)
Bréchet et al., 2021 [122]	2 AD-related dementia (ADRD)	≤ 4 mA 40 Hz HB-tACS	14 weeks, 5 days/week, single 20-min morning session/day	**Two anodes:** left AG (C1 and CP5) **Four cathodes:** C3, P2, T7 and P7	- Power spectral analysis and source localization through HD-EEG frequency modulation by presentation of autobiographical photographs - Memory and global cognitive functioning: MIS and Mini/5 min, non-visual version of MoCA) - Report of impact on daily activities [**Testing checkpoints:** at baseline, every two weeks during the 14-week intervention and every four weeks after the completion of the study for 3 months]	- Theta and gamma power increases; power decrease in the alpha and beta range; no changes in high gamma range - Better scores in both tests completed every 2 weeks as compared with baseline - Improvements in daily activities (fewer instances of repeating asking of the same questions, better orientation, greater recall of plans, etc.)	Common and mild SE (mainly sensations of tingling and burning on the electrode site)
Cappon et al., 2023 *(NCT04783350)* [123]	8 AD	2 mA 40 Hz HB-tACS	**Acute Phase** (14 weeks, 5–7 days/week, single 20-min session/day) **Hiatus Phase** (2–3 months without stimulation) **Taper Phase** (3 months, 2–3 sessions/week, single 20-min stimulation)	**Two anodes:** left AG (C1 and CP5) **Four cathodes:** C3, P2, T7 and P7	- Memory and global cognitive functioning: MIS and MoCA - Power spectral analysis and source localization through resting-state HD-EEG (only in a subset of 3 participants) [**Testing checkpoints:** at baseline and post-intervention (at the end of each of the three phases)]	- Improvement in MIS at the end of the Acute Phase, sustained during the Hiatus and Taper Phases - No changes in the MoCA at the end of the Acute Phase; modest decrease during the Hiatus and Taper Phases from baseline - Decreased theta/gamma ratio in left AG at the end of the Acute Phase	Mild SE in 25% of sessions, moderate SE in 5% and severe SE in 1% (mainly “sensations under the electrode” and “sleepiness”)
Dhaynaut et al., 2022 [120]	4 mild-to-moderate AD	≤ 4 mA 40 Hz tACS	4 weeks, 5 days/week, single 1-h session/day	Bilateral temporal lobes (T8-T7 and P7-P8)	- Power spectral analysis through resting-state EEG (recorded before and after each stimulation session and the week before and after the entire tACS treatment course) - Amyloid, p-Tau and microglia PET imaging - Cognitive assessments: ADAS-Cog, MMSE and MoCA - ADL [**Testing checkpoints:** at baseline and post-intervention]	- Weekly gamma power increased during tACS sessions but dipped over weekends; increase in gamma spectral power from baseline to post-intervention - Significant decrease of p-Tau PET in 3 subjects; significant decrease of microglial activation PET in 1 subject; Amyloid PET did not show any significant changes - No significant changes in overall cognition tests	N/D
Jacobson et al., 2022 *(NCT05203523)* [150]	75 MCI or probable early or moderate dementia (divided into *3 groups*: tACS + Exr, sham + Exr, and Exr only)	1.5 mA peak-to-peak 40 Hz tACS (simultaneously with Exr for *groups 1 and 2*)	4 weeks, 5 days/week, two 30-min sessions/day (separated by 30-min break) After an 8-week washout, the two groups are crossed over to repeat the same protocol for 4 weeks	**Active electrode:** left DLPFC **Reference electrode:** contralateral supraorbital area	- Assessments: WMS-IV; MADRS; visual 1-back task; NPI; ADAS-Cog - Virtual reality navigation; EVestG - Cerebral metabolic activity through fNIRS [**Testing checkpoints:** at baseline (week 0), post-first intervention (week 5 – after treatment – and week 11 – after washout period), post-second intervention (week 16 – follow-up), and at a long-term follow-up (week 27)]	N/D (Ongoing study)	N/D
Jones et al., 2023 *(NCT04646499)* [137]	13 amnestic MCI	1.6 mA 40 Hz tACS (simultaneously with stimulation tasks—MDT and Spot-the-Difference)	Single 1-h session/day, 5 days/week for one week (week 2), and one session/week on the same weekday for the following three weeks (weeks 3–5)	Hipp, IPL and pars triangularis with electrodes placed at 8 locations bilaterally (F7, F8, FT7, FT8, T7, T8, P7 and P8)	- Cognitive assessments: CVLT, modified version of PAT and semantic fluency task (F-A-S and animals) - Structural and functional brain network connectivity through sMRI, resting-state fMRI and DTI - Neuronal excitatory/inhibitory balance through MRS-based metrics - Blood-based biomarker detection (i.e., Aβ_40_, Aβ_42_ and Aβ_42/40_ ratio, p-Tau, NfL, GFAP) through Simoa - IADL [**Testing checkpoints:** at baseline (week 1) and post-intervention (week 5)]	- Rescued performance in MDT to that of a healthy aged-matched control group (who also conducted this task in a separate study [162]) - Significant improvement in SDFR (part of CVLT) and in F-A-S task and a non-significant “trend” for gain in the LDFR (part of CVLT) and on the PAT recall - Increase only in IPL-Hipp RSFC - No significant differences in MRS data - No significant change in Aβ, p-Tau or NfL levels - No significant change in IADL	Barely noticeable headaches, tingling, itching and phosphenes
Kehler et al., 2020 [148]	17 MCI and mild-to-moderate AD (divided into *2 groups*: tACS + Exr and Exr only)	1.5 mA peak-to-peak 40 Hz tACS (simultaneously with Exr for *group 1*)	4 weeks, 5 days/week, two 30-min sessions/day separated by 30-min break	**Active electrode:** left DLPFC **Reference electrode:** right supraorbital area	- Assessments: WMS-IV and MADRS [**Testing checkpoints:** at baseline, post-intervention (immediately after the 4 weeks of treatment) and at follow-up (1-month after post-intervention)]	- Significant improvement in WMS in both groups from baseline to post-intervention but only tACS-treated group demonstrated a better WMS score also at 1-month follow-up - Slight drop in MADRS from baseline to post-intervention (remained stable at 1 month follow-up) in the tACS-treated group	N/D
Kim et al., 2021 [144]	20 MCI (divided into *3 groups*: tACS, tDCS and sham)	2 mA for both 40 Hz tACS and tDCS	3 weeks, three 30-min sessions on the same weekday. Each participant attended three separate sessions, each alternately involving either tACS, tDCS, or sham stimulation	Same electrode placement for all experimental groups **Anode:** F3 (left DLPFC) **Cathode:** F4 (right DLPFC)	- Neuropsychological assessments: ST and TMT (A and B scores) - Power spectral analysis and source localization through resting-state EEG [**Testing checkpoints:** at baseline and post-intervention]	- Significant within-subject effect of intervention (tACS, tDCS, and sham) on changes in TMT-B scores and ST (in the latter test, the tACS group showed greater changes) - Significant within-subjects effect of intervention (tACS, tDCS, and sham) on EEG-power changes at the delta, theta, and beta frequency bands - Increased changes in activity of the anterior cingulate at the β2 frequency band in tACS group	Mild pricking sensation
Liu et al., 2022 *(NCT05251649)* [146]	1 AD	**tACS**: 1.5 mA peak-to-peak, 40 Hz **SS:** 60 dB, 40 Hz	**tACS**: 3 weeks, 5 days/week, single 20-min session/day **SS:** 3 weeks, 5 days/week, single 14-min session/day (stim. for 5-min, rest for 5-min, stim. for 5-min, rest for 1-min, and stim. for 4-min)	Two pads placed in the DLPFC and the contralateral supraorbital area	- Assessments: ADAS-Cog, MoCA, MMSE, AVLT, CDR, BAI [**Testing checkpoints:** at baseline, post-intervention (the end of the treatment for 3 weeks) and at follow-up (4 months after the intervention)]	- Improvement in overall cognitive scores at both post-intervention and 4-month follow-up - Unchanged BAI score	Self-reported anxiety symptoms, but with the BAI scale unchanged
Liu et al., 2023 *(NCT05251649)* [147]	87 mild-to-moderate AD (divided into *3 groups*: tACS + SS, only tACS and only SS)	**tACS**: 1.5 mA peak-to-peak, 40 Hz **SS:** 60 dB, 40 Hz	**tACS**: 3 weeks, 5 days/week, single 20-min session/day **SS:** 3 weeks, 5 days/week, single 14-min session/day (stim. for 5-min, rest for 5-min, stim. for 5-min, rest for 1-min, and stim. for 4-min)	Two pads placed in the DLPFC and the contralateral supraorbital area (F3 and F4)	- Assessments: CDR, ADAS-Cog, MoCA, MMSE, AVLT, BDI, HAMA, BNT, NPI - ADL - Gray matter and white matter integrity through sMRI and connectivity changes through fMRI (only at baseline and post-intervention) [**Testing checkpoints:** at baseline (before the first treatment and on the day of treatment), post-intervention (after the last treatment and on the day of treatment) and at 12-week post-treatment follow-up]	N/D (Ongoing study)	N/D (Ongoing study)
Lu et al., 2023 *(NCT05544201)* [145]	99 mild-AD (divided into *3 groups*: HD-tACS, HD-tDCS and sham HD-tCS)	2 mA for both 40 Hz HD-tACS and HD-tDCS	4 weeks, three 20-min sessions/week for both HD-tACS and HD-tDCS	Same electrode placement for all experimental groups **Anode:** F3 (left DLPFC) **Four cathodes:** around left DLPFC	- Sleep disturbances through Pittsburgh Sleep Quality Index (PSQI) - Cognitive assessments: WLLT, MoCA (Hong Kong version), ANT, CVFT - ADL - Saliva Aβ_40_ and Aβ_42_ levels [**Testing checkpoints:** 1 week before tACS (baseline, T0), after 4 weeks (T1), 8 weeks (T2), 12 weeks (T3) and 24 weeks (T4)]	N/D (Ongoing study)	N/D (Ongoing study)
Moussavi et al., 2021 [149]	28 with various degrees of dementia (divided into *2 groups*: tACS + Exr and Exr alone)	1.5 mA peak-to-peak 40 Hz tACS (simultaneously with Exr for *group 1*)	4 weeks, 5 days/week, two 30-min sessions/day	**Active electrode:** left DLPFC **Reference electrode:** contralateral supraorbital area	- Assessments: WMS-IV and MADRS - Egocentric Spatial Orientation [**Testing checkpoints:** at baseline, post-intervention (on first day of week 5) and at follow-up (1-month after post-intervention)]	- Improvement in overall cognitive scores (WMS-IV) from baseline to post-intervention for both groups but only tACS + Exr group continuing to improve even at 1-month follow-up (although it was not statistically significant) - MADRS remained unchanged throughout the sessions - Both groups showed similar improvements on spatial assessment scores in all testing checkpoints	N/D
Naro et al., 2016 [155]	35 AD, 25 MCI, 27 healthy elderly (NOLD) (divided into *2 groups*: tACS and sham)	1 mA peak-to-peak 40–120 Hz tACS	10-min session (number of sessions not reported) Each participant was randomly administered with all the conditioning protocols (receiving at least one protocol every seven days)	**tACS Group:** **Active electrode:** M1 (C3), DLPFC (AF3-AF7), DMPFC (AF3-F1), PMA (FC3), or SMA (FCz) of the left hemisphere **Reference electrode:** right mastoid **Sham Group:** **Active electrode:** M1 (C3) **Reference electrode:** C4	- Neuropsychological assessments: MMSE, RML, Verbal DS forward, CDT, AM, CVF and LVF - Power spectral analysis through resting-state EEG - ADL and IADL [**Testing checkpoints:** before (T_0_), post-intervention (T_1_) – for EEG recordings, even after 60 min (T_1+60_) – and at 2-year post-treatment follow-up (T_2_)]	**NOLD Group:** - Increase in gamma spectral power induced only by DLPFC, DMPFC and M1 tACS at T_1_ and T_1+60_, remained stable at T_2_ - Improvement in RML, DS, AM, CVF and LVF after DLPFC- and DMPFC-tACS at T_1_, remained stable at T_2_ - Gamma spectral power changes positively correlated with AM and CDT increase **MCI Group:** - Increase in gamma power in all the electrodes in 21 patients after DMPFC-tACS at T_1_ and T_1+60_, remained stable at T_2_ - No significant DMPFC-tACS after-effects at T_1_ and T_1+60_ in 4 patients, who showed an increase in gamma spectral power when compared to T_0_ and converted to AD at T_2_ - Improvement in RML, DS, AM, CVF and LVF at T_1_, remained substantially stable at T_2_ in 17 patients - Gamma power changes positively correlated with AM and CDT increase after DMPFC-tACS at T_1_ **AD Group:** - No clinical/electrophysiological effects at T_1_ and T_1+60_ - Increase in gamma spectral power, deterioration in MMSE, RML and AM at T_2_	No significant SE, including visual disturbances
Santarnecchi et al. *(NCT03880240)* [163]	55 MCI-to-moderate-AD (divided into *4 groups*: 3 tACS groups and 1 sham group; see treatment duration for details)	N/D	**Group 1 (tACS):** 2 weeks, 5 days/week, single 1-h session/day **Group 2 (tACS):** 4 weeks, 5 days/week, single 1-h session/day **Group 3 (tACS):** 4 weeks, 5 days/week, two 1-h sessions/day **Group 4 (sham):** 2/4 weeks, 5 d ays/week, once/twice 1-h sessions/day	Region with the highest brain amyloid load	- Amyloid and Tau PET imaging - Functional brain network connectivity through fMRI - Power spectral analysis through resting-state EEG; brain plasticity and markers of response to stimulation assessments through combined tACS-EEG and TMS-EEG - Cognitive assessment: ADAS-Cog - Blood and saliva collection, optional LP [**Testing checkpoints:** at baseline and post-intervention]	N/D (Ongoing study)	N/D (Ongoing study)
Sprugnoli et al., 2021 *(preliminary data for NCT03290326 and NCT03412604)* [114]	15 mild-to-moderate AD (divided into *3 groups*: see treatment duration for details)	4 mA 40 Hz tACS (simultaneously with video playback to maintain a constant brain state)	**Group 1 and 2** (5 + 5 patients): 2 weeks, 5 days/week, single 1-h session/day **Group 3** (5 patients): 4 weeks, 5 days/week, single 1-h session/day	**Group 1** (5 patients): Personalized unilateral (right) temporo-frontal lobes (based on the amyloid load distribution) **Group 2 and 3** (5 + 5 patients): Bilateral temporal lobes	- Resting-state EEG - Cognitive assessments: ADAS-Cog, MMSE, MoCA, Craft Story 21 Recall Immediate and Delayed, CVF (animals) - Cerebral perfusion through perfusion-MRI (ASL) - ADL [**Testing checkpoints:** at baseline and post-intervention]	- Increase in narrow-gamma spectral power - No significant changes in overall cognition tests - Significant CBF increase in multiple anatomical clusters, primarily in right temporal lobe, and positive correlation with narrow-gamma power changes and with changes in episodic memory and fluency in cognitive performance	Headaches, mild tingling, mild-to-moderate scalp irritation and visual changes
Sprugnoli et al., *(NCT03290326)* [114]	Estimated 20 mild-to-moderate AD (including *groups 1 and 2* in Sprugnoli et al., 2021)	4 mA 40 Hz tACS	2 weeks, 5 days/week, single 1-h session/day	Region with the highest brain amyloid load	- Amyloid PET imaging - Structural and functional brain network connectivity through sMRI and fMRI - Power spectral analysis through resting-state EEG; brain plasticity and markers of response to stimulation assessments through combined tACS-EEG - Cognitive assessment: ADAS-Cog [**Testing checkpoints:** at baseline and post-intervention]	N/D (Ongoing study)	N/D (Ongoing study)
Sprugnoli et al., *(NCT03412604)* [114]	Estimated 10 mild-to-moderate AD (including *group 3* in Sprugnoli et al., 2021)	4 mA 40 Hz tACS	4 weeks, 5 days/week, single 1-h session/day	Region with the highest brain amyloid load	- Amyloid, Tau and microglia PET imaging - Structural and functional brain network connectivity through sMRI and resting-state fMRI; cerebral perfusion through perfusion-MRI (ASL) - Power spectral analysis through resting-state EEG; brain plasticity and markers of response to stimulation assessments through combined tACS-EEG and TMS-EEG - Cognitive assessment: ADAS-Cog [**Testing checkpoints:** at baseline and post-intervention]	N/D (Ongoing study)	N/D (Ongoing study)
Xing et al., 2020 *(NCT03920826)* [121]	40 mild-AD (divided into *2 groups*: tACS and sham)	15 mA peak‐to‐peak 40 Hz tACS	Thirty 1‐hour sessions across 3 weeks	One pad placed on the forehead and two pads behind each ear (mastoid area)	- Volume and white matter integrity through sMRI - Brain connectivity and neural activity through resting-state EEG and EEG-fMRI - Amyloid PiB-PET imaging - Assessments: ADAS-Cog; MMSE, CDR‐SB, MoCA, WHO-UCLA AVLT, DS forward and backward, Geriatric Depression Scale, TMT B minus A score (B–A), BNT, NPI, ADL [**Testing checkpoints:** at baseline, post-intervention and at 3-month follow-up]	N/D (Ongoing study)	N/D (Ongoing study)
Zhou et al., 2022 [119]	50 mild-to-moderate-AD (divided into *2 groups*: tACS and sham)	2 mA 40 Hz tACS	6 weeks, 5 days/week, single 20-min session/day	Bilateral temporal lobes	- Peripheral blood Aβ_40-42_ levels through ELISA - Cognitive assessments: MMSE and ADAS-Cog [**Testing checkpoints:** at baseline (0-week), post-intervention (6-week) and after the end of tACS (end + 12-week)]	- Better MMSE score at 6-week and the end + 12-week - Better ADAS-Cog scores at 6-week but not at the end + 12-week - Aß 40:42 ratio significantly decreased only in the tACS group from baseline to 6-week - Change of Aβ 40:42 ratios significantly correlated with that of ADAS-Cog scores from baseline to 6-week	N/D

*ADAS-Cog* Alzheimer’s Disease Assessment Scale-Cognitive Subscale, *ADCS* Alzheimer’s Disease Cooperative Study, *ADL* Activities of daily living, *AG* Angular gyrus, *AM* Attentive matrices, *ANT* Attention network test, *ApoE* Apolipoprotein E, *ASL* Arterial Spin Labelling, *AVLT* Auditory Verbal Learning Test, *BAI* Beck Anxiety Inventory, *BDI* Beck Depression Inventory, *BDNF* Brain-Derived Neurotrophic Factor, *BNT* Boston Naming Test, *CBF* Cerebral blood flow, *CBI* Caregiver burden inventory, *CDR* Clinical Dementia Rating Scale, *CDR‐SB* Clinical Dementia Rating Scale Sum of Boxes, *CDT* Clock Drawing Test, *CVF* Category verbal fluency, *CVLT* California Verbal Learning Test, *DLPFC* dorsolateral prefrontal cortex, *DMPFC* Dorsomedial prefrontal cortex, *DS* Digit span, *DTI* Diffusion tensor imaging, *EEG* Electroencephalography, *ELISA* Enzyme-linked immunoassay, *ESO* Egocentric spatial orientation, *EVestG* Electrovestibulography, *Exr* Cognitive exercises, *fMRI* functional magnetic resonance imaging, *fNIRS* functional near-infrared spectroscopy, *FNAT* Face-name associations task, *GDS* Global deterioration scale, *GFAP* Glial fibrillary acidic protein, *HAMA* Hamilton Anxiety Scale, *HB* Home-based, *HD* High-definition, *HD-EEG* High-density electroencephalography, *Hipp* Hippocampus, *IADL* Instrumental activities of daily living, *IPL* Inferior parietal lobe, *LDFR* Long-delay free recall, *LP* Lumbar puncture, *LVF* Letter verbal fluency, *M1* Primary motor cortex, *MADRS* Montgomery-Åsberg Depression Rating Scale, *MDT* Mnemonic Discrimination Task, *MIS* Memory Index score, *MMSE* Mini-Mental State Examination, *MoCA* Montreal Cognitive Assessment, *MRI* Magnetic resonance imaging, *MRS* Magnetic resonance spectroscopy, *NfL* Neurofilament light, *NPI* Neuropsychiatric Inventory Questionnaire, *PAT* Paired-associates task, *PET* Positron emission tomography, *PiB* Pittsburgh Compound B, *PMA* Premotor area, *Pz* Precuneus, *RAVLT* Rey Auditory Verbal Learning Test, *RML* Reversal motor learning, *RSFC* Resting-state functional connectivity, *SAI* Short-latency afferent inhibition, *SDFR* Short-delay free recall, *SE* Side effects, *SMA* Supplementary motor area, *SS* Sound stimulation, *ST* Stroop Test, *sMRI* structural magnetic resonance imaging, *tCS* transcranial current stimulation, *TMS* Transcranial magnetic stimulation, *TMT* Trail-Making Test, *WHO-UCLA AVLT* World Health Organization-University of California-Los Angeles Auditory Verbal Learning Test, *WLLT* Word-List Learning Test, *WMS* Wechsler Memory Scale, *ZBI* Zarit Burden Interview

## Current knowledge of gamma tACS for the treatment of AD

### Pre-clinical studies

A reduction in adult hippocampal neurogenesis is considered a remarkable hallmark of AD [89]. Neurogenesis throughout adulthood occurs only in specific brain regions, primarily in the subventricular zone of the lateral ventricles and the subgranular zone of the hippocampal dentate gyrus, and plays an important role in hippocampus-dependent learning and memory, exploratory behaviour and pattern recognition [90, 91]. The different neurogenesis stages – like proliferation, differentiation, survival and maturation of newborn neurons – are affected to varying degrees in AD and can thus be partially responsible for the cognitive deficits and hippocampal atrophy [89, 92]. Many studies have therefore assessed whether different strategical interventions, including brain stimulation with ultrasound, magnetic waves, as well as deep brain electrical stimulation, can improve cognition in AD *via* the enhancement of adult neurogenesis [93, 94]. One study, in fact, investigated whether intracranial alternating current stimulation (IACS) at 40 Hz – a modified version of tACS – could stimulate hippocampal neurogenesis in the 5 × FAD mouse model of AD [95]. The authors showed that 40 Hz IACS could increase the number of cells expressing the neurogenesis markers Ki67, Nestin and doublecortin in both hippocampal dentate gyrus and subventricular zone of 5 × FAD mice [95], suggesting the potential of this technique to enhance adult neurogenesis, particularly considering that such markers are linked to the normal migration of neurons into the cerebral cortex.

Whether or not the facilitation of adult neurogenesis in 5 × FAD mice can directly result in functional improvements in memory and learning was not investigated by the same authors, but an independent work confirmed that 40 Hz tACS in 5 × FAD mice could significantly enhance the LTP of synaptic responses in the CA1 region of the hippocampus [96]. LTP is a fundamental synaptic plasticity process associated with the strengthening of neural connections, crucial for memory consolidation and learning, and its potentiation following 40 Hz tACS is a powerful indication that the technique can be promising for functional improvements in the memory defects affecting AD patients. Yet, the mechanisms related to the enhanced LTP following 40 Hz tACS in 5 × FAD mice are not yet understood. For example, the tACS protocol did not change the levels of key neuroplasticity-related proteins such as brain-derived neurotrophic factor (BDNF, an important growth factor that regulates synaptic plasticity, neurogenesis and neuronal survival), cAMP response element-binding (CREB) and its phosphorylated form pCREB (well-known transcription factors that play crucial roles in modulating gene expression associated with synaptic plasticity, learning and memory; [96]). The lack of changes in BDNF, CREB and pCREB levels suggests that the effectiveness of 40 Hz tACS in enhancing LTP might engage alternative neurobiological mechanisms within the brain. It is indeed possible that the effect of tACS is linked to changes in the functioning of pre- or post-synaptic neurons, that could be related to neuronal excitability, glutamate release probability or efficacy of post-synaptic receptors, in relation to the gamma entertainment mediated by the stimulation, which allows the enhancement of communication between excitatory neurons [97]. Otherwise, the enhanced LTP might be the result of enhanced excitability and plasticity of adult-born neurons [95], given the typical high input resistance and high resting potentials of newborn cells [98–100]. Although the mechanisms underlying the modulation of synaptic plasticity by 40 Hz tACS remain fairly elusive, different stimulation frequencies applied *via* tACS have shown similar cognitive-improving and plasticity-enhancing effects. For instance, alpha-tACS stimulation in the visual cortex has demonstrated after-effects, likely attributed to STDP [39, 40, 101, 102] and, similarly, NMDAR-mediated STDP has been proposed as the mechanism of plastic changes upon beta-tACS in the motor cortex [103]. Of note, stimulation at 40 Hz frequency with techniques different from tACS also induces long-term plasticity changes that are phase-sensitive. For example, Wespatat et al. (2004) highlighted the phase-dependency of synaptic modifications, revealing NMDA receptor-dependent LTP during peak-pairing and NMDA receptor-independent LTD during trough-pairing [104]. Additionally, Wang et al. (2023) showed that 40 Hz optogenetics stimulation, but not 10-Hz stimulation, facilitates gamma oscillations and rescues synaptic plasticity deficits induced by stroke, *via* phase-locking the activity of PV-INs during peak phases, and enhancing the activity of pyramidal neurons during the trough phases of gamma oscillations [105]. This underscores how the phase relationship between pre- and post-synaptic activity critically influences synaptic plasticity, in line with the fundamentals of Hebbian learning theory.

Notably, 40 Hz tACS was also shown to be effective in enhancing the LTP-like plasticity induced by intermittent theta-burst stimulation applied to the primary motor cortex or the dorsolateral prefrontal cortex (DLPFC) [106, 107]. This enhancement of LTP by theta-gamma coupling is linked to an enhancement of the gamma power and to modifications in short-interval intracortical inhibition, which serves as a neurophysiological marker for GABA_A_ receptor activity [106]. These findings imply that gamma tACS may directly influence GABAergic neurotransmission within cortical regions, potentially leading to sustained alterations such as continuous enhancement of gamma band activity. Therefore, while the exact relationship between tACS frequency and its effects on synaptic plasticity remains to be studied, the literature suggests that entraining synchronous oscillations, particularly at the gamma frequency range, can positively modulate synaptic plasticity and facilitate cognitive processes such as attention, memory and learning. Given that dysfunctions of cognitive processes can in part result from altered or disrupted gamma neuronal oscillations, the potential therapeutic applications of tACS become a valuable asset in the context of AD. However, in order to clarify the mechanisms of action of this technique, future pre-clinical studies with 40 Hz tACS should better analyse the electrophysiological effects of the stimulation, focusing on basal synaptic transmission, glutamate release, functions of AMPA and NMDA receptors and excitability of pyramidal and GABAergic neurons.

Another interesting aspect deriving from pre-clinical experiments on animal models of AD is the effect of 40 Hz tACS on pathophysiological markers such as Aβ and neuroinflammation. A recent study, aimed at comparing different tACS protocols to better evaluate the effects of short- and long-term stimulations (7 days *versus* 21 days) in APP/PS1 mice, revealed that long-term 40 Hz tACS treatment increases spontaneous gamma power, enhances cross-frequency coupling and improves memory performance in the Y-maze compared to sham stimulation, indicating functional enhancements [108]. Importantly, long-term tACS reduced Aβ accumulation and changed microglia morphology in the hippocampus. This is in strict accordance with the fact that gamma entrainment by 40 Hz optogenetics or visual stimulation reduces Aβ *via* phagocytosis by microglia [69], but additional experiments are necessary to better understand how 40 Hz tACS acts on amyloid turnover, and whether it acts through decreased amyloidogenesis or increased amyloid endocytosis by phagocytic microglia. Indeed, authors only showed a reverse in microglial morphology, with a reduction of the cell body diameter in mice with long-term tACS treatment compared to short-term treatment or sham treatment [108]. For this reason, it would be interesting to accurately demonstrate the specific effects and the underlying biological mechanism of tACS on Aβ clearance.

While the study on long-term tACS demonstrated that the efficacy of gamma tACS is related to the duration of the stimulation session [108], most recent data have also highlighted the need to pay attention to the stimulation intensity of the tACS protocol. In fact, a study applying IACS at 40 Hz [109] confirmed the ability of this technique to reduce the Aβ load, improve memory and influence the morphology and activation state of microglia in the cortex and hippocampus of 5 × FAD mice, but these improvements were more evident when a highest current intensity was applied (200 μA).

**Fig. 2:**
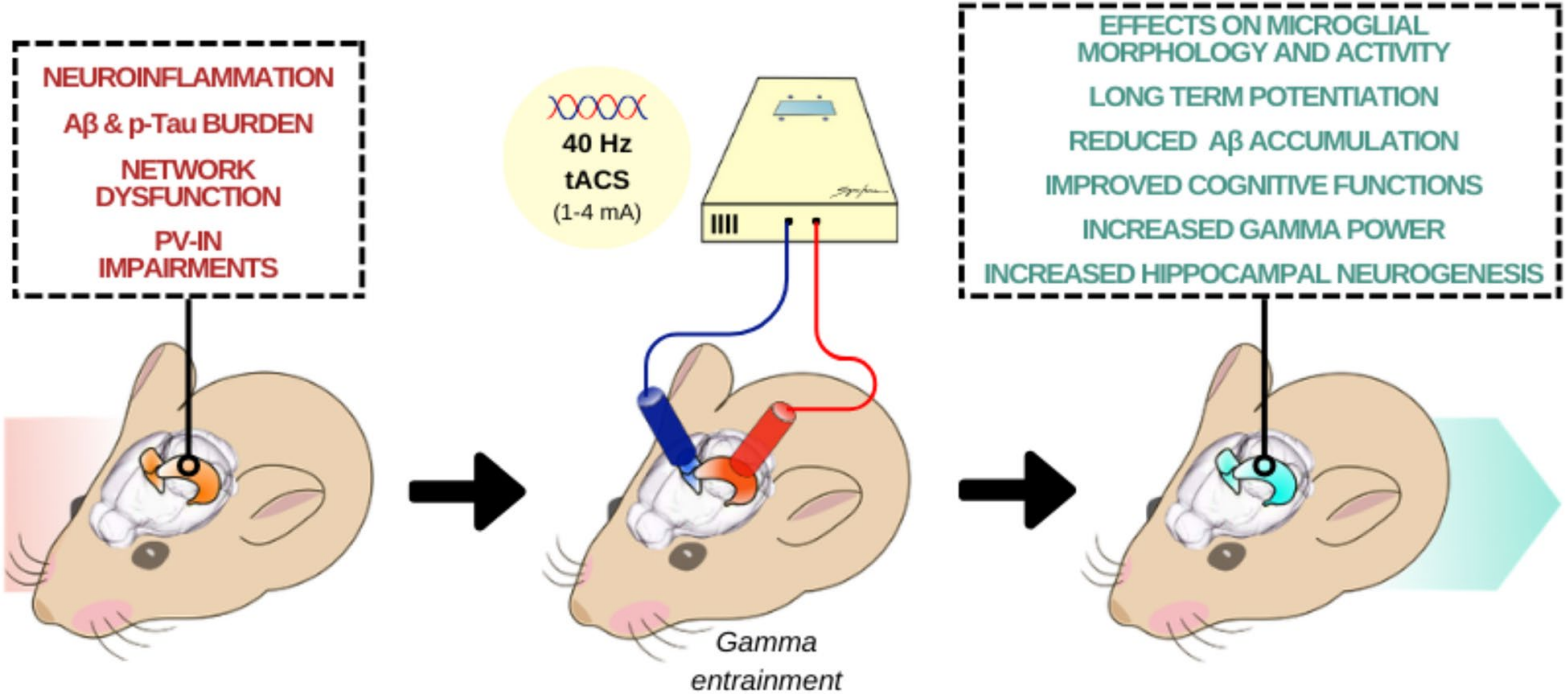
Cognitive impact of 40 Hz tACS in the AD murine hippocampus. The portrayal of the murine model of AD provides insights into the typical pathological features of AD, with a focus on the hippocampus (brain region pivotal for memory and cognition, commonly implicated in the early stages of the disease). The murine hippocampus exhibits significant signs of neuroinflammation, Aβ and tau accumulation, highlighting compromised neuronal networks and PV-IN activity. Subsequently, 40 Hz tACS reveals a series of improvements in terms of reduction in Aβ accumulation, enhanced cognitive functions, increased gamma power, and promotion of adult hippocampal neurogenesis. These outcomes suggest that 40 Hz tACS might positively impact brain physiology, delineating the connection between 40 Hz stimulation and the observed positive effects in the murine AD hippocampus, and offering fundamental insights into its potential therapeutic relevance for human AD. However, further exploration of other implicated brain regions and/or mechanisms involved is imperative to comprehend the holistic effects of 40 Hz tACS on AD pathology

Thus, in this pre-clinical frame of exploring gamma tACS in AD treatments, animal studies are still limited, likely due to the technical and methodological challenges related to electrode implantation, duration of the treatment protocol and intensity of the current delivery. Moreover, there are concerns about translational compatibility of tACS results across species and techniques [110]. Indeed, in a translational comparison, the correlation between animal findings and humans should be carefully run [51]. Since most of the in vivo experiments in AD mice were conducted in anesthetized or immobilized animals, it should be considered that these manipulations can change network functions and alter neural dynamics and brain metabolism compared to the awake, freely moving state [111]. In fact, tACS could yield different results on neural activity, even when the same stimulation protocol is used, because the electrophysiological responses also depend on the current state of the brain. In addition, voltage gradients and current intensities described in animal research cannot be directly compared to human investigations. Disparities in brain volume, anatomy and skull thickness can all have a significant impact on the physiological effects of the technique [51]. Moreover, only a few AD mouse models have been used so far, and for this reason a deeper and expanded research is necessary to strengthen the translational relevance and validity of 40 Hz tACS in AD. For example, computational models or in vivo whole-brain imaging could be successful tools for understanding which regions are the most influenced by the different stimulation paradigms, what is the proportion of stimulation diffusion and which regions are unaffected by a particular electrode montage [112, 113]. Fortunately, despite these limitations (e.g., limited behavioural testing, short treatment periods, small sample sizes, potential anaesthesia and stress-induced side effects), the pre-clinical outcomes described above offer a promising scenario for a more detailed evaluation of the therapeutic potential of gamma tACS to block or at least slow down AD cognitive decline (Fig. 2).

### Clinical studies exploring 40 Hz tACS

Many clinical studies have evaluated the feasibility and efficacy of 40 Hz tACS in MCI and AD patients, by testing different electrode placements, durations and modalities of treatment and/or stimulation intensities. Some of these studies have also analysed the possibility of exploiting tACS devices at home (home-based 40 Hz tACS studies; HB-tACS). In this section, we will discuss the clinical trials that have been published so far using 40 Hz tACS, including ongoing trials showing either preliminary results or exclusively the study design (see summary in Table 1).

### 40 Hz tACS over the bilateral temporal lobes

Bilateral temporal stimulation is being applied in ongoing trials ([114] – clinical trials NCT03290326 and NCT03412604). The targeted areas were selected based on the results of amyloid positron emission tomography (PET) imaging showing the maximal tracer uptake in these regions. In particular, the pilot clinical experiment (trial NCT03290326) aims to test the safety, feasibility and effectiveness of 40 Hz tACS in reducing the Aβ load, modulating EEG gamma-band spectral power and improving cognitive abilities. The second interventional trial (trial NCT03412604), in order to produce a longer-lasting effect, has evaluated the tolerability and effectiveness of a stronger stimulation protocol by assessing brain connectivity, cognitive functions and cerebral brain flux (CBF). The authors showed that gamma spectral power changes were correlated with CBF increase, as well as with moderate improvements in cognitive performance related to episodic memory and fluency. As a matter of fact, AD is characterized by reduced brain metabolism [115, 116], and for this reason, an increase of blood flow after treatment constitutes a very promising biomarker of tACS efficacy. Indeed, it has been recently demonstrated that gamma activity can modulate blood vessel diameter in the human brain, although the underlying mechanisms have not been clarified yet [58]. Of note, the improvements in cognitive functions [114] are in line with the crucial role of the bilateral temporal lobes in many cognitive abilities (i.e., facial recognition, communication and processing of emotions; [117]). Moreover, the temporal cortex represents a brain area where gamma band dysfunctions can be detected with whole-head magnetoencephalography in AD patients [118]. Importantly, another study with bilateral temporal lobe gamma tACS [119] not only showed a reduction of the plasma Aβ_42_/Aβ_40_ ratio but also an improvement in Mini-Mental State Examination (MMSE) and AD Assessment Scale cognitive subscale (ADAS-Cog) scores immediately after 6 weeks of stimulation. This may be related to the restoration of gamma oscillations in the target area, with a functional enhancement of the connected circuits. However, the effects were not seen in the 12-week follow-up, probably because the benefits have a limited duration, as the exogenous electrical stimulation can transiently entrain the endogenous gamma waves but is not long enough to induce permanent changes. In line with this hypothesis, in another study the 40 Hz tACS protocol was delivered on temporal regions only for 4 weeks, a sufficient period to see a decrease of p-tau burden and microglial activation using PET imaging with selective tracers ([^11^C]-PiB, [^18^F]-FTP and [^11^C]-PBR28, respectively), but without any changes in Aβ levels and in overall cognition [120]. This is also in accordance with the first study mentioned above [114] in which a maximum of 4 weeks of gamma tACS did not show significant improvements in global cognition. Overall, these studies suggest that the duration of stimulation protocol has a vital importance in showing long-lasting outcomes.

Finally, an ongoing double-blind, randomised controlled trial on mild-AD patients using 40 Hz tACS aims at targeting indirectly the temporal lobes by the usage of three electrodes, one placed on the forehead and the other two on each mastoid area, as the current flows through the bilateral frontal and temporal lobes. Their aim is to evaluate neural plasticity using structural and functional MRI (sMRI and fMRI, respectively) in addition to other traditional biomarkers and clinical parameters [121] (clinical trial NCT03920826; see also Table 1).

### 40 Hz tACS over the parietal lobes

In other studies ([122, 123], clinical trial NCT04783350), researchers stimulated a specific area of the lateral parietal cortex, the left angular gyrus, in which the conscious experience of retrieving vivid, rich and multi-sensory episodic memories resides [124–126]. Interestingly, both groups used an HB-tACS protocol that was administered by a caregiver previously receiving a lab-based training, while the adherence was remotely supervised in real-time by physicians. The authors found an improvement in the Memory Index Score (MIS), a measure of delayed recall memory. In both studies, the stimulation determined gamma power increase analysed by high-density electroencephalography (HD-EEG). Although Bréchet et al. (2021) only enrolled two patients and thus the results should be considered extremely preliminary, this study represents an important and emblematic pilot assessment of the feasibility of HB-tACS. In Cappon’s group instead, despite the memory improvement in MIS, the participants only showed modest benefit in the Montreal Cognitive Assessment (MoCA) test. According to the authors, this result is likely due to the fact that the MIS evaluates both the free recall and the cued recall condition, while the MoCA only measures the delayed free recall condition and thus a larger effect can be reached by using MIS [123]. Despite the limited number of patients involved, these studies suggest that the left angular gyrus may be a suitable target area for specific spatial modulation of brain gamma oscillations to induce memory enhancement. In addition, both works demonstrate the high adherence, feasibility and safety of remotely monitored, caregiver-administered HB-tACS to improve memory in the elderly people. Indeed, home participation can overcome accessibility problems and increase recruitment, allowing for the implementation of larger and longer clinical trials while preserving clinical standards and high-quality research.

Other groups are testing HB-tACS protocols [127, 128] (clinical trials PNRR-POC-2022–12376021 and NCT05643326, respectively), to also evaluate plasma levels of neurofilament light chains (NfL), astrocyte markers (GFAP) and Aβ_42_/Aβ_40_ ratio. From a functional point of view, they evaluated a battery of neuropsychological tests, the short-latency afferent inhibition (SAI), an intracortical excitability transcranial magnetic stimulation (TMS) parameter referring to the cholinergic neurotransmission, and the resting-state functional magnetic resonance imaging (rs-fMRI) connectivity. The targeted area in this study is the precuneus, a medial portion of the parietal lobe playing a pivotal role in different brain networks including the default mode network, a “brain network node” frequently damaged in AD [129]. The precuneus is involved in different cognitive abilities such as visuo-spatial imagery, episodic memory retrieval, self-processing and consciousness [130].

Although the aforementioned trials are still ongoing and there are no published results yet, Benussi and colleagues [131] (clinical trial NCT04515433) targeted the precuneus in MCI-due-to-AD patients, showing that gamma tACS is able to restore SAI *via* a mechanism that is, however, still unclear. Furthermore, the preliminary results of this trial [131] showed that patients had improved performance in the Rey Auditory Verbal Learning test (in both immediate and delayed recall) and in the face-name associations task, related to associative and episodic memory retrieval [132, 133]. Since alterations in the default mode network are already detectable in MCI and are crucial for the disease development [134, 135], the precuneus could represent a suitable and accessible target that can be precociously stimulated to slow down the network degeneration. A subsequent study by the same authors extended the previous results in a larger number of AD patients, indicating a possible positive effect of precuneus-tACS on memory, confirming that these improvements are related to gamma entrainment, as demonstrated by EEG recordings [136] (clinical trial NCT04842955).

Another ongoing study focuses on amnestic MCI patients using tACS combined with mnemonic tasks to engage episodic memory [137] (clinical trial NCT04646499). It also evaluates plasma AD biomarkers and neuroplastic changes through rs-fMRI connectivity and neuronal excitation/inhibition (E/I) balance. The latter can be analysed by non-invasive proton magnetic resonance spectroscopy (MRS) to determine the concentrations of brain metabolites such as GABA and glutamate/glutamine ratio, which are used as a measure of neuronal E/I ratio. In fact, previous studies support a relationship between functional connectivity and regional excitability [138, 139], and hyperexcitability is already diagnosed since the MCI phase [27–29]. Indeed, MCI and AD patients exhibit deficits in these neurotransmitters [140, 141]. In this context, tACS could represent a valid tool to restore E/I alterations and brain hyperexcitability. In conclusion, these preliminary results arising from parietal lobe stimulation are promising in restoring some of the cognitive functions usually impaired in AD, and future data may corroborate the current state of art.

### 40 Hz tACS over the DLPFC

Another major hotspot of the default mode network is the DLPFC, frequently targeted in clinical trials as it is highly affected by Aβ deposition and constantly impaired in AD patients [142, 143]. Some studies have focused on comparing tDCS with 40 Hz tACS ([144, 145]– clinical trial NCT05544201). Other studies have evaluated the effects of acoustic co-stimulation ([146] – clinical trial NCT05251649; [147] – clinical trial NCT05251649) or coupled tACS with cognitive exercises ([148–150] – clinical trial NCT05203523).

The first study listed above [144] showed differences between 40 Hz tACS and tDCS in MCI patients. Gamma tACS significantly improved performance in higher-level cognitive functions, in contrast to tDCS, although both induced changes in different brain waves. For example, gamma tACS increased beta-frequency activity, but surprisingly, had no effect on gamma activity, suggesting that 40 Hz tACS did not appear to entrain endogenous gamma oscillations in the mentioned study. Nonetheless, the stimulation protocol could induce plastic changes within the stimulated area or network, allowing cognitive restructuring and neuroplasticity since the MCI stages. Indeed, the MCI brain compared to the AD brain is still more plastic and shows more capacity for improvements, confirming the importance of a precocious treatment. In an ongoing trial, another research group is evaluating the effectiveness of 40 Hz high-definition tACS (HD-tACS) compared to HD-tDCS delivered on the same area [145] (clinical trial NCT05544201). Since tDCS shows positive effects on post-sleep declarative memory in AD patients [151–153], they are also examining the potential effect of tACS treatment in improving sleep-related brain activity and quality [145]. They expect to find better outcomes using HD-tACS as it is more effective in triggering the endogenous slow gamma oscillations and enhancing brain functions in healthy individuals as well as in patients with different brain disorders [154].

Interesting data also originate from studies combining 40 Hz tACS with 40 Hz sensory stimulation, driven by the initial results on pre-clinical AD [69]. In a case study, the acoustic stimulation at 40 Hz in combination with 40 Hz tACS had a long-lasting effect on cognition, visual perception and attention in a 74-year-old AD patient [146]. To validate the outcomes, the same authors designed a specific protocol on patients with mild and moderate AD comparing the same paired stimulation pattern (acoustic and electrical) with tACS or sound stimulation alone [147]. As in the case report, participants are expected to show improvements not only immediately after the stimulation but also after a 4-month follow-up. This can be presumably explained by the fact that tACS has timely effects in improving cognition, and the combination of tACS and sensory stimulation could have “offline effects” on neuroplastic processes that could occur both during the treatment and after the end of the stimulation. In the first pilot study [146], the authors revealed an increase in MMSE and MoCA scores of the patient after a 4-month follow-up and a reduction in ADAS-Cog. This is an intriguing aspect as it would be interesting to evaluate with cyclic and scheduled follow-ups the maximum temporal limit in which the effects are still detectable. In another study, the potential longer-lasting effect of 40 Hz tACS on the left DLPFC, coupled with cognitive exercises delivered through a tablet app, was explored [148]. The researchers aimed at strengthening cognitive functions by emphasizing on the spatial orientation, associative memory, word-image association, visual memory and categorization [148]. The cognitive status of all participants improved, yet only the group that received both exercises and the 40 Hz tACS continued to improve at the 1-month follow-up, indicating that tACS might potentially induce sustained enhancements when coupled with cognitive training. Later, the same group confirmed the previous results, with patients choosing whether to combine the cognitive exercises with repeated 40 Hz tACS or performing the exercises alone. They proved that tACS coupled with brain exercises is more beneficial and effective for performance in cognitive and spatial tests [149]. However, since in this study the placebo effect was not examined, researchers are complementing their work through a specialized, crossover, double-blind, placebo-controlled trial [150].

### 40 Hz tACS as a potential screening tool

Finally, it is worth mentioning a pioneer study [155] aimed at focusing on the differences in the tACS effects between MCI and AD patients. tACS was delivered in the full spectrum of gamma bands (i.e., 40 to 120 Hz) to stimulate distinct areas of the left hemisphere. The authors found that tACS did not have a modulatory effect on AD patients when compared to MCI and age-matched healthy volunteers. Interestingly, some of the MCI patients were responsive to the tACS protocol by showing an increased gamma power and improvements in neuropsychological tests, while others did not; in particular, the unresponsive patients to tACS converted to AD two years later. Thus, the authors suggested that, besides the therapeutic potential, gamma-tACS could be employed as an early screening tool for identifying MCI individuals with high risk of dementia and conversion to AD. This is also in line with the neuroimaging results, which confirm that functional changes precede structural alterations. During the MCI period, in fact, brain anatomy remains relatively intact, while synaptic dysfunction begins to impact dynamic connectivity. At this stage, activation of the residual neural reserve networks plays a crucial role in preserving normal brain functions. Consequently, neurophysiological methods might be well-suited to detect early alterations, especially in the MCI brain [156]. Because of this, the 40 Hz tACS – which was first developed as an effective tool to treat MCI/AD – might conceal an unexplored potential also for screening the risk of conversion from MCI to AD. Thereby, this technique may emerge as a promising “theranostic” approach, having both diagnostic and therapeutic potential. In fact, tACS may not only be an innovative treatment for AD prevention in people with MCI, but may also be a good tool for identifying MCI patients at high risk of developing AD. Indeed, Naro et al., 2016 (see also [156]) suggested that such patients are unresponsive to tACS treatment [155]. In this context, it would be informative to couple tACS with EEG recordings following stimulation, as the confirmation of persistent after-effects following tACS may provide indications for the effectiveness of the treatment.

### Non-40 Hz frequency performance

It is evident that the predominant focus of investigations in the realm of tACS for MCI or AD has been on the specific frequency of 40 Hz. Notably, none of these studies have explored alternative frequencies to establish a control condition. Instead, they have uniformly opted for 40 Hz stimulation, with the exception of Naro et al. (2016), who employed continuous and random stimulation spanning from 40 to 120 Hz, thus encompassing the entire gamma frequency range [155]. However, this study did not include non-40 Hz gamma frequencies as control conditions but instead utilized sham stimulation, consistent with other clinical studies cited in the current review.

As previously argued, it is worth noting that the specific choice of 40 Hz was initially influenced by the study of Iaccarino et al. (2016), who demonstrated that flicker stimulation at 40 Hz reduced Aβ levels in a mouse model of AD, with no effects observed when mice were exposed to other stimulation parameters or frequencies (e.g., constant light-on or darkness, flicker at 20 Hz or 80 Hz and random flicker), suggesting a frequency-dependent neuromodulatory effect of the stimulation [69]. Similarly, optogenetic stimulation of PV-INs at other frequencies (e.g., 8 Hz or random noise) had no effect on the induction of gamma oscillations [157, 158], or on the levels of Aβ [69] in AD mice, in line also with the notion that entrainment is effective when the stimulating frequency resembles the endogenous frequency of the targeted cells.

40 Hz GENUS has exhibited promising outcomes even in cognitively healthy adults. Notably, PET scans have revealed a distinctive pattern of regional CBF enhancement during binaural auditory stimulation set at 40 Hz, with this frequency proving to be the most effective among the 12 stimulation frequencies tested. This increase in EEG activity amplitude at the 40 Hz steady state has been correlated with heightened cortical activity [159], emphasizing a specific response to gamma sensory stimulation. Additionally, Santarnecchi et al. (2013) investigated various frequencies (40 Hz, 5 Hz, non-periodic high-frequency tRNS and no stimulation) of prefrontal tACS in healthy individuals, demonstrating that tACS at the specific frequency of 40 Hz led to a notable decrease in the time needed to solve complex logic problems [160]. In a subsequent study however, the authors found that the efficacy of prefrontal 40 Hz stimulation varied based on individual capabilities, with those displaying slower baseline performance benefiting more [161]. The authors cautioned against directly linking cognitive performance and stimulation efficacy, as the study participants were relatively homogeneous in terms of baseline performance and age. Thus, generalizing these results to diverse populations, such as those with varying ages or initial cognitive deficits, would be premature. Nevertheless, the study suggested that individual differences in frequency responsiveness, such as reduced endogenous prefrontal gamma activity in slower responders, may influence tACS effects. Therefore, since reduced gamma activity is a common feature in AD, it is plausible to assume that individual differences in frequency responsiveness, as observed in the study, may differentiate the effects of 40 Hz tACS in subjects with MCI or AD, depending on the level of impairment of gamma activity.

This does not negate the need for further studies to specifically investigate the efficacy of other tACS frequencies besides 40 Hz in AD. In fact, while the sham-controlled studies have provided valuable insights into the potential benefits of 40 Hz tACS in MCI and AD populations, the lack of alternative frequency stimulations as control conditions limits our understanding of the specificity and efficacy of gamma frequency stimulation. Given the complex nature of neural oscillations and the variability in individual responses to tACS, it is essential to employ a range of control conditions to disentangle the unique effects of 40 Hz stimulation from nonspecific factors. Thus, moving forward, future studies should consider the inclusion of varied control conditions to provide a more comprehensive understanding of the therapeutic potential of tACS and, more generally, NIBS.

## Conclusions and future perspectives

tACS is a promising tool to challenge AD, particularly for its ability to selectively modulate brain oscillations with substantial spatial and frequency specificity. This has allowed researchers to target different brain regions using gamma waves that are closely associated with memory and cognitive functions notably affected in AD. Within the broad spectrum of gamma band frequencies, the low gamma at 40 Hz is particularly significant. This growing interest in the usage of 40 Hz tACS is supported by the promising results observed in AD mouse models. Pre-clinical investigations suggest that gamma tACS may exert beneficial effects on neurogenesis, synaptic plasticity, cognitive performances and pathological hallmarks of AD, such as Aβ levels and neuroinflammation. Such findings push researchers and scientists to investigate the potential therapeutic implications also in patients. Available data obtained from clinical trials suggest that gamma tACS can reduce p-tau, Aβ and microglial activation and ameliorate clinical outcomes related to learning and memory processes. In particular, MCI and/or AD patients show improvements in episodic, autobiographical, working, declarative and associative memory, amongst others. Interestingly, gamma tACS is administered in distinct areas (such as the DLPFC, precuneus and left angular gyrus, among others) with positive results, showing the crucial impact of these different brain areas in disease evolvement and highlighting the complexity of the pathology.

40 Hz tACS is employed to target the main pathological hallmarks, but since AD is considered a “disconnection syndrome” [26, 118, 164], tACS is primarily used to address circuit dysfunction, mitigating the potential propagation of these alterations throughout the brain. Although the exact mechanism is not completely known – and hence pre-clinical studies are crucial – it appears that 40 Hz tACS initially influences the stimulated brain region at a local level, modulating and synchronizing its gamma rhythms. With the reset of local gamma waves, it is then possible to restore the coordination and communication of the brain regions that are functionally connected to the targeted area, ultimately leading to a systemic effect, resulting in the amelioration of AD hallmarks and symptomatology.

**Fig. 3:**
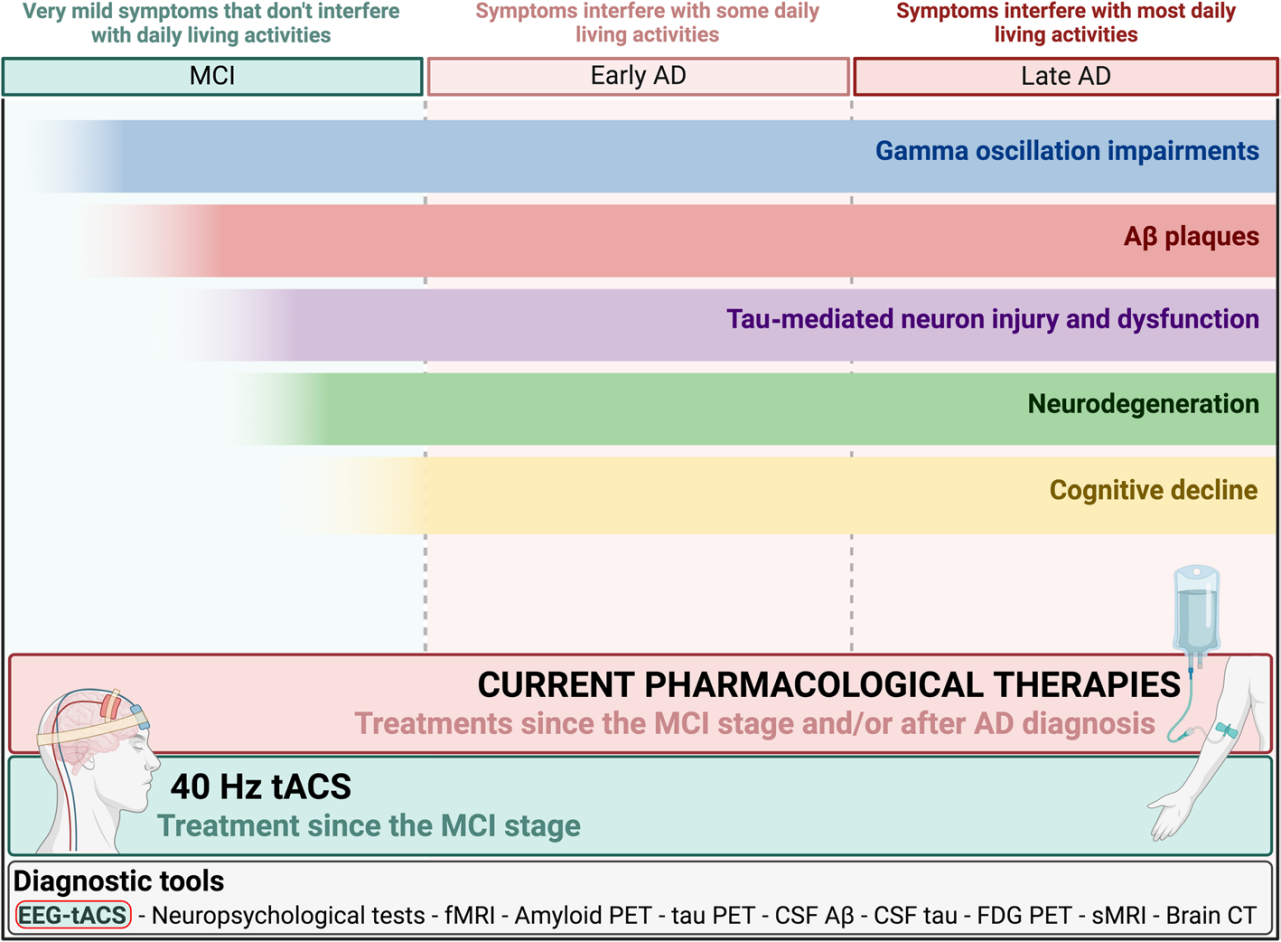
Spectrum of AD: biomarker trends and intervention options. The gradient bars describe the manifestation of the key hallmarks across the AD continuum, in which symptom severity increases throughout all the disease stages. Specifically, one of the earliest detectable aberrations include gamma wave disturbances, followed by significant plaque deposition, tau-mediated lesion processes and neurodegeneration, while cognitive function is relatively preserved in the early stages. Importantly, due to its safety and feasibility, 40 Hz tACS can be administered as early as the MCI stage to restore gamma waves, thus providing a key opportunity to modulate all other biomarkers. Similarly, the FDA-approved lecanemab can be administered early, during MCI and early AD to reduce the amyloid load. This time window provides a critical advantage for early treatment, and the combined usage of both approaches (tACS and lecanemab) might be highly beneficial. Alternatively, tACS can be applied alone, when patients are not eligible for pharmacological treatment. Of note, early treatment requires early diagnosis; in this framework, compared to the current diagnostic tools, EEG-tACS could represent an ideal strategy for future prompt intervention

The implications of using 40 Hz tACS are straightforward. Since neural disconnection is already detectable from the early disease stage, tACS can be precociously used in the MCI phase or even before, when brain damage can still be restored, blocked and/or delayed. Indeed, the disease acts in a subtle way for 10–15 years without symptoms, then it becomes mildly symptomatic and finally it manifests severely. In the latter stage all the “neural reserve” has been completely exhausted and even the most effective treatment (pharmacological or not) is likely to fail. Therefore, intervention during the initial phases of cognitive impairment may offer a paramount advantage, when neural reserve is relatively intact, and the disease is still at the beginning [165]. In this scenario, the application of tACS alone, or in combination with monoclonal antibody treatment such as lecanemab, approved for patients with MCI and early AD [14], can be highly beneficial when the intervention occurs at the earliest appearance of cognitive decline. In the same line of thinking, tACS can be essential when the patient is not eligible for the monoclonal antibody therapy, thus making tACS an attractive, alternative solution (Fig. 3).

Another clear indication deriving from the usage of 40 Hz tACS, and indeed other NIBS techniques offering equally promising results against AD, is the need to focus beyond the amyloid targeting. This is particularly evident from yet another recent failure of a phase-3 trial, in which the anti-Aβ antibody solanezumab, tested in pre-clinical AD patients with high brain amyloid levels, failed to slow clinical decline despite a lower increase in amyloid levels after 240 weeks of treatment [166].

In this context, timely diagnosis has a decisive role and is key to identifying the best window of clinical intervention. Together with fMRI and PET, in recent years, EEG and its derived HD-EEG, have gained increasing importance in early and accurate AD diagnosis. The analysis of EEG brain signals may provide valuable insights into the functional alterations in the brain circuitry, while cognitive impairment progresses. These techniques, complemented by machine-learning algorithms, allow the identification of subtle alterations and pinpoint the exact location of circuitry dysfunction before severe clinical symptoms burst out [165, 167]. In a future perspective, in fact, it would be useful to combine EEG (or HD-EEG) with 40 Hz tACS, to specifically and directly stimulate those areas that have been previously detected as dysfunctional (see for example [168]; Fig. 3). Interestingly, a similar approach has already been developed using TMS, with the research team employing TMS-EEG to determine the precise location of brain oscillation disruptions and assess the immediate effects of stimulation [169]. Unfortunately, an important limit for routinely using tACS-EEG is the presence of recording artifacts. These artifacts not only hinder the adaptation process, especially for the target frequency, but also act as interference in observing the true effects of 40 Hz tACS on the brain’s neural activities. In recent years, several research laboratories have been developing specific methods to remove the artifact from EEG recordings for concurrent EEG signal acquisition during tACS [168, 170–172].

Overall, although 40 Hz tACS has shown positive outcomes, sometimes it also yields contradictory results. While some patients have shown substantial and persistent outcomes, others showed poor effects only limited to the stimulation period and some did not show any improvements at all. This marked disparity raises questions regarding the replicability and the longevity of the tACS treatment and justifies the need of additional and more personalised studies. To fully exploit the potential of 40 Hz tACS in the context of AD, a multifaceted approach is therefore needed. First, future research should prioritize pre-clinical experiments aimed at unravelling the effects of 40 Hz stimulation, with an emphasis on electrophysiological, molecular and cellular effects related to the improvements observed in AD models, and on the underlying mechanisms involved. In the clinical setting, instead, researchers should work to define more precise and personalised stimulation protocols. For example, 40 Hz tACS has been shown to improve cognitive symptoms in major depressive disorder patients or to attenuate symptoms in patients with obsessive compulsive behavior [173, 174]. Consequently, since AD patients are affected by both cognitive and neuropsychiatric symptoms, clinical settings could take advantage of tACS effects to restore these alterations. Thus, identifying and tailoring tACS to the specific symptomatology of the patient is important for the individualization of a personalized therapy. In this regard, given its ease of use, convenience and cost-effectiveness, 40 Hz tACS is also a valuable tool for home-based treatment with very limited side effects, but strict patient adherence protocols should be exercised to guarantee efficacy and reproducibility.

In conclusion, 40 Hz tACS represents an evolving technique in designing multidimensional intervention therapies (both alone and in combination with other types of treatment). Therefore, given its flexibility and effectiveness, the medical community can exploit 40 Hz tACS benefits to revolutionize the AD treatment landscape.

BOX 1 Reduction of gamma oscillations in AD The reduced power of gamma oscillations in AD is believed to be caused by a redistribution of the synaptic drive between active and silent neurons, with reductions of the inhibitory tone followed by disinhibition of firing in active cells [23, 175]. In fact, there is a strong correlation between the number of hyperexcitable cortical cells and brain Aβ load, with hyperactive neurons identified near Aβ plaques [176]. A reduction of the GABAergic inhibitory terminals on cortical neurons proximal to Aβ plaques can be observed both in humans and in animal models [177]. This is due to a functional impairment of inhibitory INs, resulting in a decline of GABA_A_ receptor currents and a redistribution of the inhibitory and excitatory drive within neuronal circuits [24, 178]. For example, in mice with the *APOE* ε4 allele, representing one of the main genetic risk factors for AD, there is a loss of GABAergic interneurons (INs) in the hippocampus [179]. Together with a general depletion of the inhibitory drive, specifically the number of PV-INs is reduced both in AD patients and animal models, in parallel with an impaired PV-IN activity, leading to disrupted brain network and hypersynchrony [68, 180]. Verret et al. (2012) identified that both AD patients and hAPPJ20 transgenic mice show PV-IN and inhibitory synaptic dysfunctions, which result in aberrant gamma oscillatory activity and cognitive alterations [67]. Similar deficits in PV-IN number or function were also detected in other AD models [180–184]. Since many factors can be involved in this network imbalance, the exact mechanisms that underlie  these deficits in PV-INs are still unknown; nevertheless, soluble Aβ forms could play a critical role in this intricated scenario, contributing to this network breakdown [68]. Interestingly, using the APP23xPS45 model, researchers found that in the hippocampal CA1 region many neurons are hyperactive before plaque formation in 1–2 month-old mice. Indeed, hippocampal hyperactivity can be induced by application of soluble Aβ in mice and it can be rescued in APP23xPS45 mice by acute treatment with a γ-secretase inhibitor, which reduces the levels of soluble Aβ in the brain [185–187]. Another important contribution in desynchrony comes from neuroinflammation that correlates with Aβ plaque progression [188]. With an overexpression of IL-1β, tumour necrosis factor alpha and IL-6, there is an increase of seizure severity that can in turn enhance inflammatory processes causing downstream cognitive effects such as inhibition of hippocampal LTP and neuronal death [189]. In addition, neuroinflammation and microglia play a fatal role in dismantling the perineuronal nets (PNNs), one of the most crucial elements in neuronal trophism and survival. PNNs are condensed extracellular matrix structures that surround neurons, particularly PV-INs. Since PV-INs have a fast-spiking activity that makes them more susceptible to deterioration, PNNs are fundamental for their correct functionality, as they create a protective “scaffold” that envelops the INs, supporting their high energy demands [190, 191]. It has been demonstrated that PNNs are reduced in AD, as a consequence of microglial activation and its proinflammatory products [191, 192]. When PNNs are degraded the protective shield is removed, and neurons are exposed to neurotoxic insults such as Aβ, leading to cell damage and death [193]. These amount to a positive feedback loop of microglial activation, PNN loss, Aβ-accumulation and neuroinflammation [194]. This vicious circle increases the brain hypersynchrony and the consequent aberrant neuronal activity which, in turn, boost all the aforementioned pathological processes, enhancing the circuit breakdown. Nonetheless, the fact that aberrant gamma activity and cognitive dysfunction in AD patients and mouse models appear at early disease stages is a valid indication that hyperactivity represents the initial step in the pathophysiological cascade and the consequent abnormalities in gamma waves.

BOX 2 Gamma oscillatory activity and the role of PV-INs PV-INs are involved in gamma rhythms in many cortical and subcortical brain structures [195], including the hippocampus, where they play a crucial role in memory processes and in higher cognitive functions [196]. Although PV-INs make up a small percentage of the total hippocampal neuronal population, about 2.6% of the total neurons and 24% of the GABAergic neurons in the CA1 region [197, 198], they play a critical role in the hippocampal network as they coordinate and stabilize pyramidal neuron communication [199], synchronizing their cortical activity during cognitive processes [62, 200, 201].

**Fig. 4:**
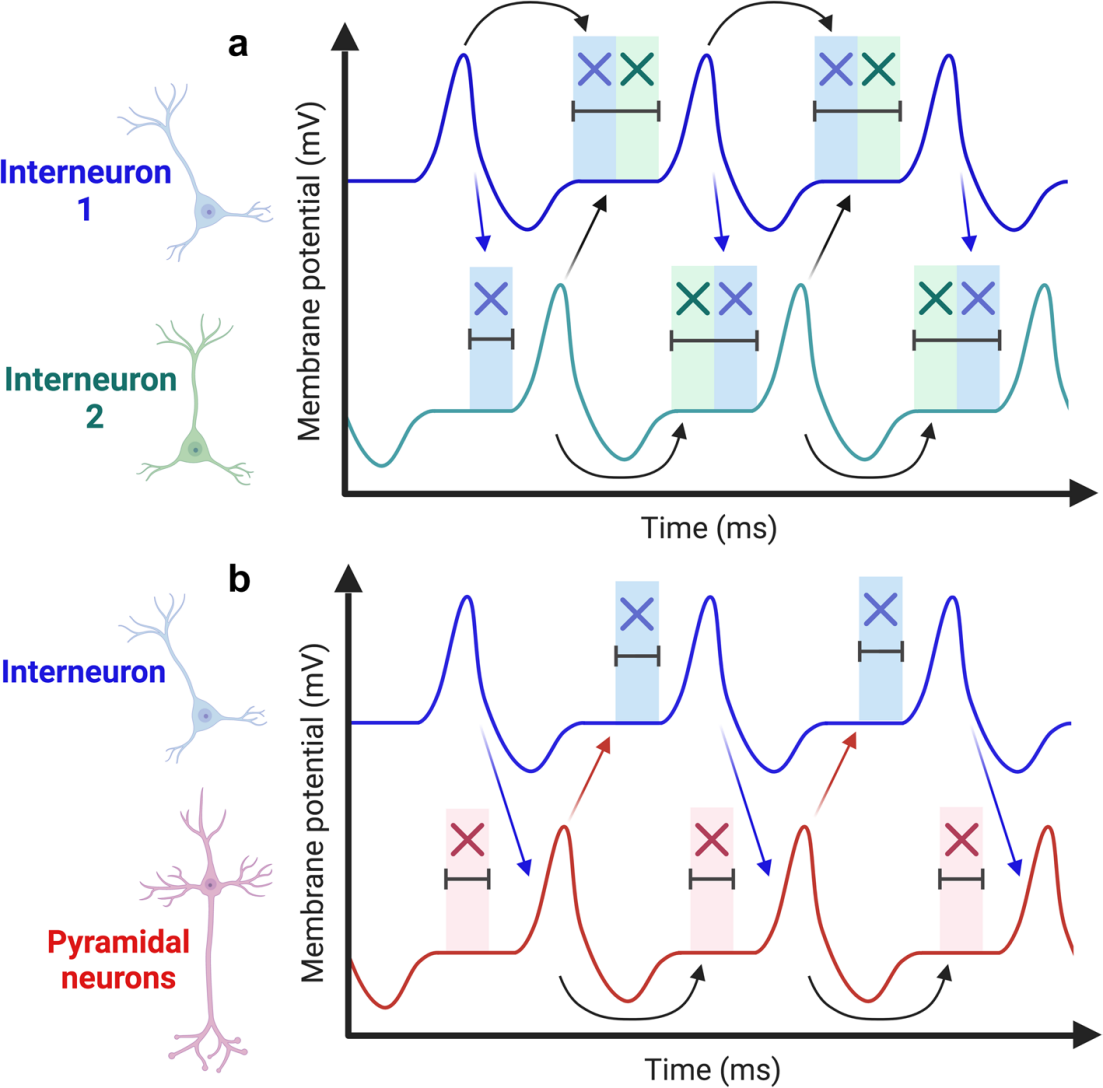
Mechanisms of large-scale gamma band synchronization in neural networks. The large-scale gamma band synchronization represents a powerful mechanism for integration and coordination of distant neurons. The firing of spatially distant neurons might show considerable delays due to axonal conduction and synaptic transmission. However, the relative phase of gamma oscillations in separated or within the same areas is cancelled, as in reality the firing of distant neurons does not show delay or is proximately zero (zero lag-phase synchrony). This synchronization mechanism is based on two main models, the first termed Inhibitory-Inhibitory (I-I), and the second Inhibitory-Excitatory (I-E). **a** The I-I model only consists of inhibitory interneurons (INs, IN1 marked blue and IN2 marked green), that are mutually inhibited *via* GABA_A_ receptors to quickly actualize zero-phase synchrony. When the first PV-IN fires, it can inhibit itself and also inhibit distant INs (blue bands). Likewise, the inhibition of the second PV-IN inhibits itself and the first PV-IN (green band). **b** The E-I model consists of excitatory pyramidal neurons (red) and inhibitory INs (blue). The short millisecond delay between the firing of the pyramidal neuron and the spike of the IN is consistent with monosynaptic excitation of the IN, which in turn activates a GABA_A_ receptor-mediated inhibition of the pyramidal cell (red band), that precedes pyramidal cell depolarization. In this way, the rhythmic activity of INs synchronizes the spiking of pyramidal cells, creating a window for action potential initiation in pyramidal cells. In this scenario, the fast excitation and the delayed feedback inhibition alternate, creating a cyclic oscillating trend that persists over time

The first evidence of PV-IN involvement in gamma oscillations came from the correlative study between PV-IN spikes and locally recorded gamma oscillations in the hippocampus of awake rats [202], showing the occurrence of PV-IN spikes always immediately after the spikes of neighbouring pyramidal neurons [62, 203]. The short millisecond delay between the firing of the pyramidal neuron and the spike of the PV-IN is consistent with monosynaptic excitation of PV-INs, which in turn activates a GABA_A_ receptor-mediated inhibition of the pyramidal cell, that precedes pyramidal cell hyperpolarization [62, 203–205]. In this way, the rhythmic activity of PV-INs synchronizes the spiking of pyramidal cells by delineating a very narrow window for action potential initiation in pyramidal cells. In this scenario, the fast excitation and the delayed feedback inhibition alternate, creating a cyclic trend that persists over time (Fig. 4). In vivo, several optogenetic studies provided the causal evidence of gamma wave evocation or silencing through cell-type-specific modulation of PV-INs [157, 158]. Additionally, gamma oscillations can also be evoked ex vivo by various agonists of metabotropic or ionotropic receptors present on these neurons [206–210].

In parallel to this Excitatory-Inhibitory (E-I) relationship that explains the rhythmic appearance of gamma waves, an Inhibitory-Inhibitory model (I-I) also exists, which only consists of inhibitory INs that are mutually inhibited *via* GABA_A_ receptors to quickly actualize zero-phase synchrony and can receive tonic or stochastic inputs (Fig. 4) [211]. Regarding the tonic drive, INs can spike with a well-defined periodicity, synchronizing their firing [212]. Conversely, when INs receive different inputs at an asynchronous state, instead of generating random fluctuations, they do not remain stable but turn synchronous over time [213–218]. In biological systems, the two models coexist and intertwine, creating a far more complex and intrigued plot in which PV-INs receive multitude number of inputs at the same time and fire following an oscillatory pattern.

This is also because of the fast-spiking nature of PV-INs and their specific biological and electrophysiological properties, as their somatic input–output relationship is about 5 times steeper compared to principal neurons [219], leading them to an increased sensitivity toward different input changes. Importantly, Kriener et al. (2022) showed that the steepness of the input–output relationship of hippocampal PV basket cells is different depending on whether they are stimulated on the soma or dendrites [220]. Indeed, the PV-IN soma is more sensitive to different amounts of input, whereas the dendrites reduce the amplitude of fast-fluctuating synaptic responses, reducing the variability in the interspike interval, thus generating the rhythmicity of gamma oscillations. The studies of biophysical properties revealed that the robustness of gamma oscillations is mainly mediated by dendrites through high-threshold and fast-activating K^+^ currents *via* Kv3-type channels [221–223], that can tone down spatial and temporal input heterogeneities and thereby enhance spike synchrony in the gamma range. To summarize, PV cells can level out different excitatory and inhibitory inputs, synchronizing at a common frequency rate, thus enhancing the robustness of gamma oscillations.

## Data Availability

Not applicable.

## Abbreviations

Aβ: Amyloid β
AD: Alzheimer’s Disease
ADAS-Cog: Alzheimer's Disease Assessment Scale-Cognitive Subscale
APP: Amyloid precursor protein
ARIA: Amyloid-related imaging abnormalities
BDNF: Brain-derived neurotrophic factor
CBF: Cerebral brain flux
CREB: CAMP response element-binding
DLPFC: Dorsolateral prefrontal cortex
E/I: Excitatory/inhibitory ratio
EEG: Electroencephalogram
fMRI: Functional MRI
GENUS: Gamma entrainment using sensory stimuli
HB-tACS: Home-based 40 Hz transcranial alternating current stimulation
HD-EEG: High-density electroencephalography
HD-tACS: High-definition transcranial alternating current stimulation
I-E: Inhibitory-excitatory
I-I: Inhibitory-inhibitory
IACS: Intracranial alternating current stimulation
LTD: Long-term depression
LTP: Long-term potentiation
MCI: Mild cognitive impairment
MIS: Memory Index Score
MMSE: Mini-Mental State Examination
MoCA: Montreal Cognitive Assessment
MRS: Magnetic resonance spectroscopy
NfL: Neurofilament light chains
NIBS: Non-invasive brain stimulation
pCREB: Phosphorylated cAMP response element-binding
PET: Positron emission tomography
PNN: Perineuronal net
PV-INs: Parvalbumin interneurons
rs-fMRI: Resting-state functional magnetic resonance imaging
SAI: Short-latency afferent inhibition
sMRI: Structural MRI
STDP: Spike-timing dependent plasticity
tACS: Transcranial alternating current stimulation
tDCS: Transcranial direct current stimulation
TMS: Transcranial magnetic stimulation
tRNS: Transcranial Random Noise Stimulation
